# Supplementary material for: Mitochondrial retrograde signaling through UCP1-mediated inhibition of the plant oxygen-sensing pathway
Source: Curr Biol. 2022 Mar 28;32(6):1403–1411.e4. doi: 10.1016/j.cub.2022.01.037 (PMC8967405; doi:10.1016/j.cub.2022.01.037)
Supplement: Document S1. Figures S1–S4 [file mmc1.pdf]

Current Biology, Volume *32*

Supplemental Information

Mitochondrial retrograde signaling through UCP1-mediated inhibition of the plant oxygen-sensing pathway

Pedro Barreto, Charlene Dambire, Gunjan Sharma, Jorge Vicente, Rory Osborne, Juliana Yassitepe, Daniel J. Gibbs, Ivan G. Maia, Michael J. Holdsworth, and Paulo Arruda

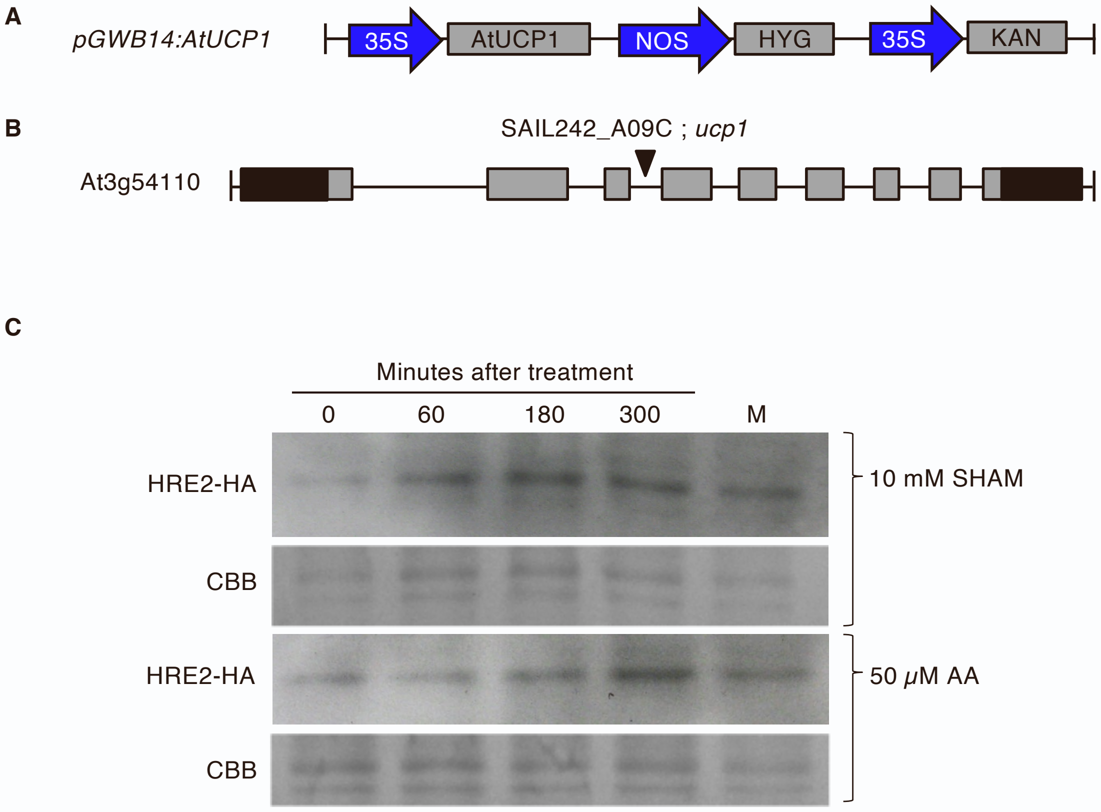

Figure S1. Diagrammatical representation of *35S:UCP1* construct transformed into *A. thaliana*, and *ucp1* mutant, and influence of mitochondrial inhibitors on stability of HRE2^{3xHA}. Related to Figure 1.
A. The *UCP1* coding sequence was recombined into the PGWB14 plasmid vector under the control of the 35S constitutive promoter and transformed into *35S:MC-HAGUS* line. **B.** A SAIL insertional knockdown for UCP1 (At3g54110) (*ucp1*). Grey boxes indicate coding regions, black untranslated regions of transcripts, blue arrows indicate promoters. **C.** Western blot analysis of HRE2^{3xHA} abundance following treatment with SHAM or Antimycin A (AA), M indicates mock samples sprayed with 2% ethanol only. CBB, Coomassie Brilliant Blue loading control.

A

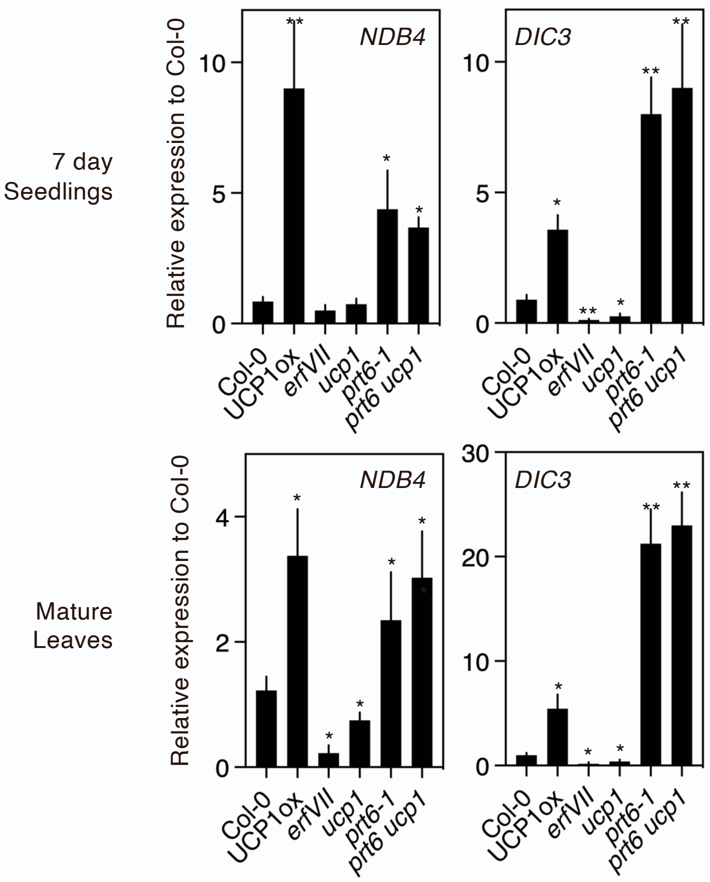

B

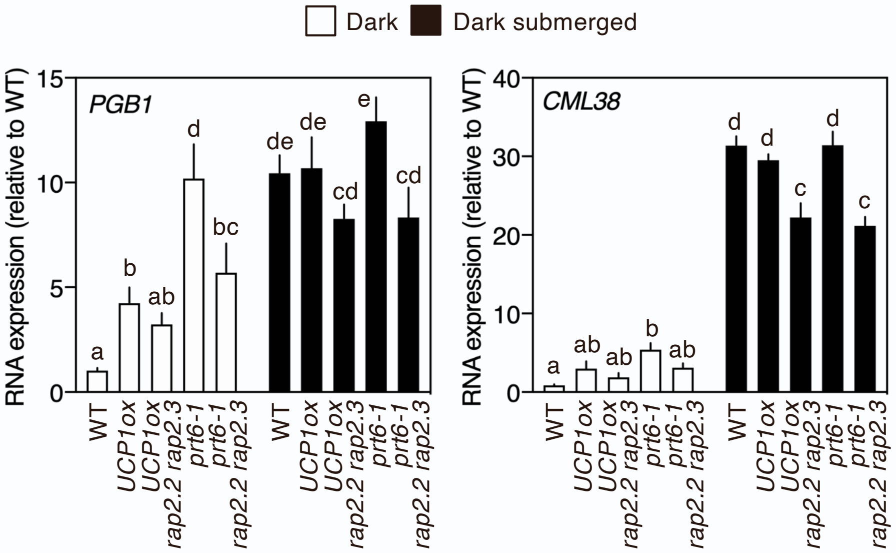

Figure S2. Expression of canonical genes of mitochondrial retrograde signalling and hypoxia response in seedlings/leaves and dark/dark+submergence. Related to Figure 2.

A. RNA expression for 7-day-old light grown seedlings for mitochondrial marker transcripts from nuclear genes *NDB4* and *DIC3*. T-tests were applied with comparison to Col-0 in each case because a large variance in gene expression was observed due to the discrepant profile of the genotypes analysed, hampering genotype differentiation by multiple comparison tests. * $p < 0.05$, ** $p < 0.01$.

B. RNA expression for 7-day-old seedlings grown in long days in control (1 h dark) or 1 h submerged+dark for WT, mutants and UCP1ox combinations.

Error bars indicate SD, letters one-way ANOVA, Tukey's test.

Figure S3, Related to Figure 3.

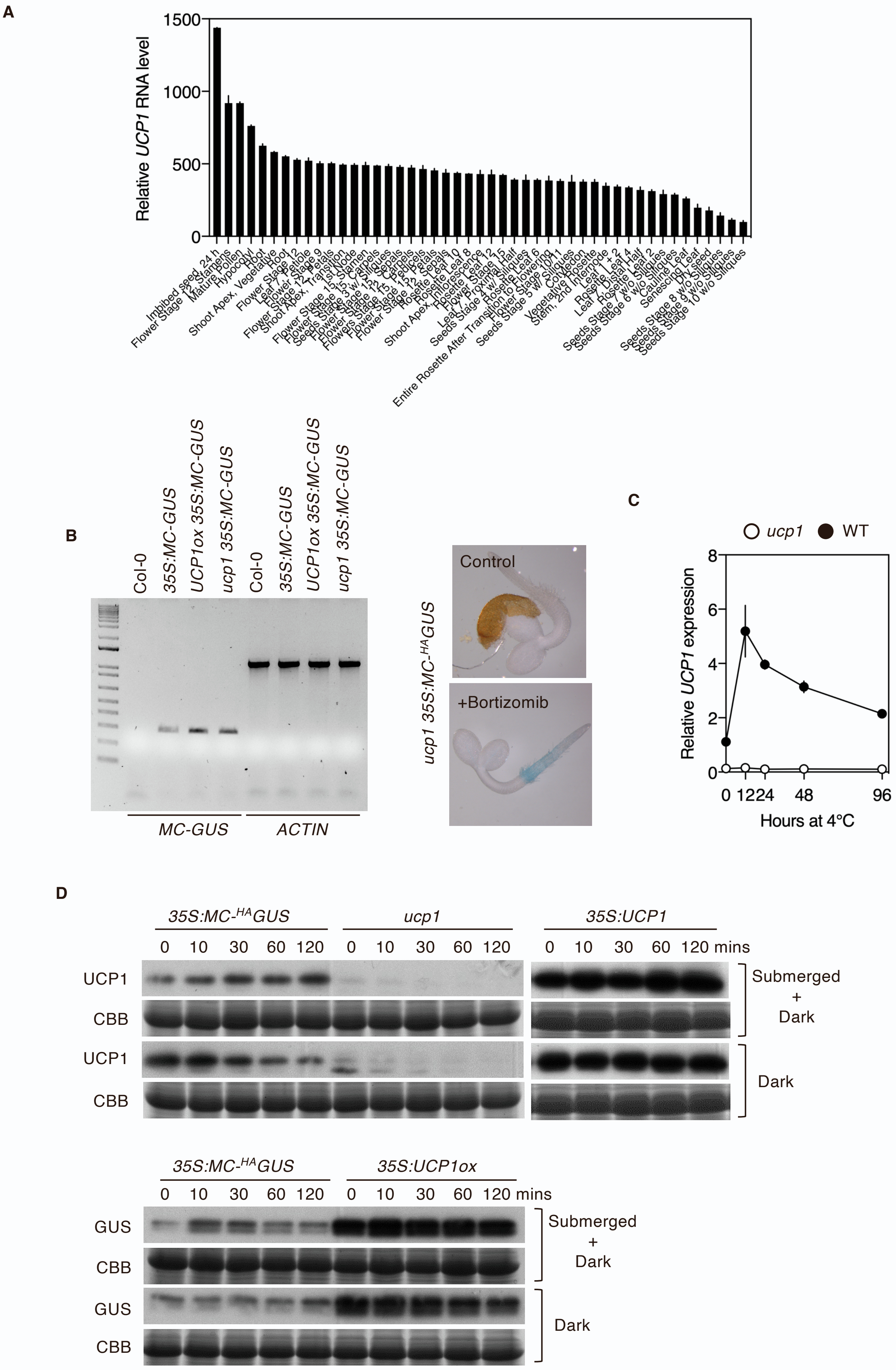

Figure S3. Gene expression and UCP1/^{HA}GUS stability. Related to Figure 3.

A. Relative levels of *UCP1* RNA expression during development in *A. thaliana* (data obtained from Bio Array Resource).

B. Semi quantitative rtPCR analysis of *MC-GUS* and *ACTIN* RNA expression in seedlings of mutant combinations and histochemical staining of GUS activity in *ucp1* seedlings in the absence (control) or presence of Bortezomib (20 mM treatment for 3h) .

C. Expression of *UCP1* RNA in WT (Col-0 *35S:MetCys-^{HA}GUS*) and *ucp1* following transfer to cold (4C).

D. UCP1 and ^{HA}GUS protein accumulation in *35S:Met-Cys-^{HA}GUS*, *UCP1ox* and *ucp1* subjected to 0, 10, 30, 60 and 120 min of submergence in water in the dark or only in the dark. CBB Coumassie Brilliant Blue.

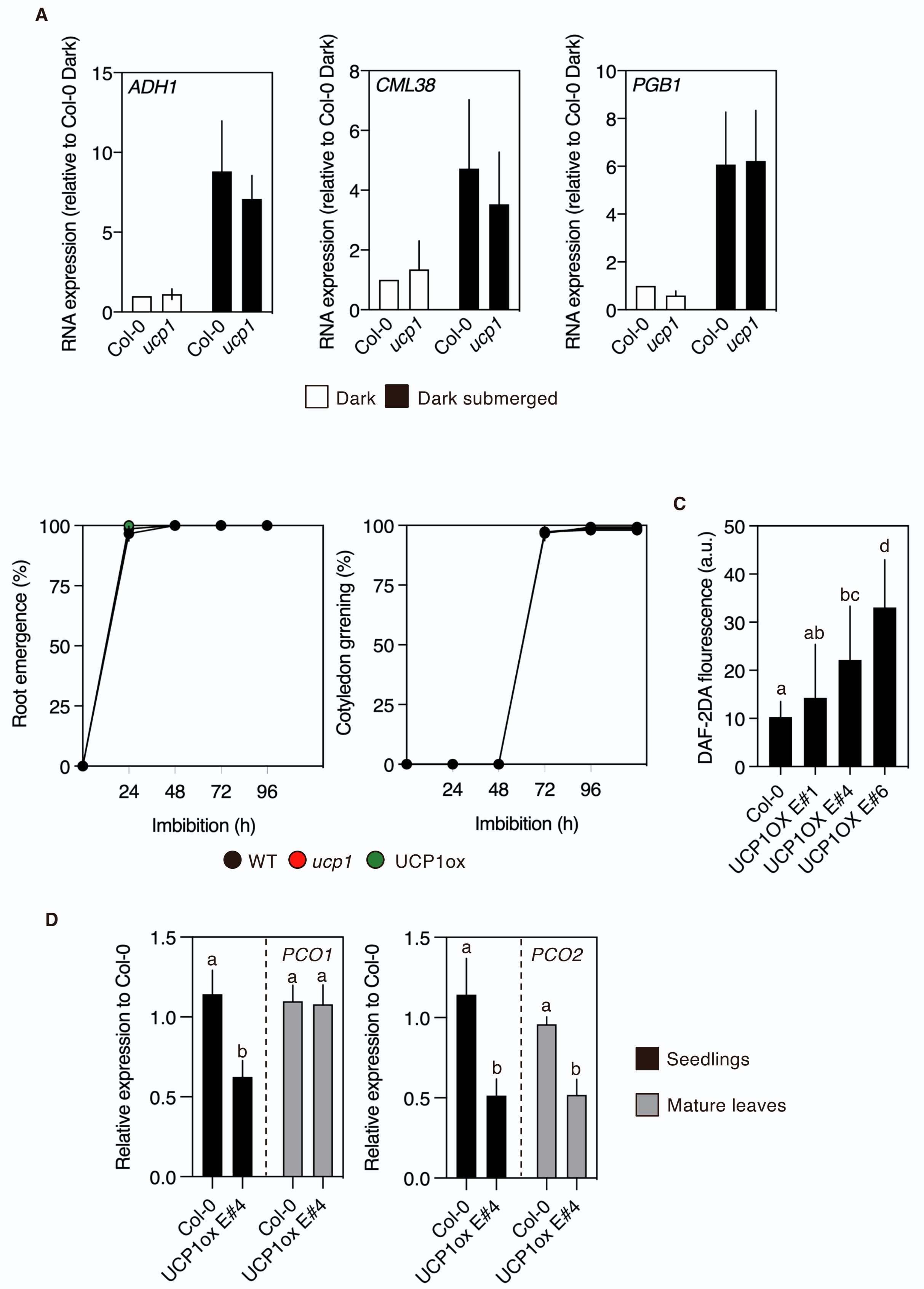

Figure S4. Gene expression, germination and NO accumulation. Related to Figure 4.
A. RNA expression for 7-day-old seedlings grown in continuous light in control (1 h dark) or 1 h submerged+dark for WT and *ucp1*.
B. Germination (root emergence) and establishment (cotyledon greening) of WT, *ucp1* and UCP1ox on 1/2MS media without ABA. Error bars indicate SD.
C. NO levels (measured as DAF-2DA fluorescence, arbitrary units) for WT and UCP1ox transgenic lines. Error bars indicate SD, letters one-way ANOVA, Tukey's test.
D. Expression of transcripts for *PCO1* and *PCO2* in seedlings or mature leaves of Col-0 and UCP1ox.
