# Supplementary material for: Mitochondrial retrograde signaling through UCP1-mediated inhibition of the plant oxygen-sensing pathway
Source: Curr Biol. 2022 Mar 28;32(6):1403–1411.e4. doi: 10.1016/j.cub.2022.01.037 (PMC8967405; doi:10.1016/j.cub.2022.01.037)
Supplement: Document S2. Article plus supplemental information [file mmc2.pdf]

Current Biology

Report

Mitochondrial retrograde signaling through UCP1-mediated inhibition of the plant oxygen-sensing pathway

Highlights

- UCP1 inhibits the PCO branch of the PRT6 N-degron pathway
- Inhibition leads to substrate stabilization and altered gene expression
- Inhibition transduces UCP1 function during development and in response to stress

Authors

Pedro Barreto, Charlene Dambire, Gunjan Sharma, ..., Ivan G. Maia, Michael J. Holdsworth, Paulo Arruda

Correspondence

michael.holdsworth@nottingham.ac.uk (M.J.H.),
parruda@unicamp.br (P.A.)

In brief

Barreto et al. uncover a retrograde signaling mechanism that links inner mitochondrial membrane protein UCP1 to cytoplasmic oxygen sensing. UCP1 activity is transduced via inhibition of the PCO N-degron pathway to influence gene expression. This mechanism integrates mitochondrial and nuclear functions during development and in response to stress.

Barreto et al., 2022, Current Biology *32*, 1403–1411
March 28, 2022 © 2022 The Authors. Published by Elsevier Inc.
https://doi.org/10.1016/j.cub.2022.01.037

CellPress

Current Biology

CellPress
OPEN ACCESS

Report

Mitochondrial retrograde signaling through UCP1-mediated inhibition of the plant oxygen-sensing pathway

Pedro Barreto,[1] Charlene Dambire,[2] Gunjan Sharma,[2] Jorge Vicente,[2] Rory Osborne,[3] Juliana Yassitepe,[4] Daniel J. Gibbs,[3] Ivan G. Maia,[1] Michael J. Holdsworth,[2,7,8,9,*] and Paulo Arruda[4,5,6,7,*]

[1]Departamento de Ciências Químicas e Biológicas, Instituto de Biociências de Botucatu, UNESP, Botucatu 18618-970, SP, Brazil
[2]School of Biosciences, University of Nottingham, Loughborough, Leicestershire LE12 5RD, UK
[3]School of Biosciences, University of Birmingham, Edgbaston B15 2TT, UK
[4]Genomics for Climate Change Research Center, Universidade Estadual de Campinas, Campinas 13083-875, SP, Brazil
[5]Departamento de Genética e Evolução, Instituto de Biologia, Universidade Estadual de Campinas (UNICAMP), 13083-862 Campinas, SP, Brazil
[6]Centro de Biologia Molecular e Engenharia Genetica, Universidade Estadual de Campinas, Campinas 13083-875, SP, Brazil
[7]These authors contributed equally
[8]Twitter: @N_end_rules
[9]Lead contact
*Correspondence: michael.holdsworth@nottingham.ac.uk (M.J.H.), parruda@unicamp.br (P.A.)

https://doi.org/10.1016/j.cub.2022.01.037

SUMMARY

Mitochondrial retrograde signaling is an important component of intracellular stress signaling in eukaryotes. UNCOUPLING PROTEIN (UCP)1 is an abundant plant inner-mitochondrial membrane protein with multiple functions including uncoupled respiration and amino-acid transport[1,2] that influences broad abiotic stress responses. Although the mechanism(s) through which this retrograde function acts is unknown, overexpression of UCP1 activates expression of hypoxia (low oxygen)-associated nuclear genes.[3,4] Here we show in *Arabidopsis thaliana* that UCP1 influences nuclear gene expression and physiological response by inhibiting the cytoplasmic PLANT CYSTEINE OXIDASE (PCO) branch of the PROTEOLYSIS (PRT)6 N-degron pathway, a major mechanism of oxygen and nitric oxide (NO) sensing.[5] Overexpression of UCP1 (UCP1ox) resulted in the stabilization of an artificial PCO N-degron pathway substrate, and stability of this reporter protein was influenced by pharmacological interventions that control UCP1 activity. Hypoxia and salt-tolerant phenotypes observed in UCP1ox lines resembled those observed for the PRT6 N-recognin E3 ligase mutant *prt6-1*. Genetic analysis showed that UCP1 regulation of hypoxia responses required the activity of PCO N-degron pathway *ETHYLENE RESPONSE FACTOR* (*ERF*)*VII* substrates. Transcript expression analysis indicated that UCP1 regulation of hypoxia-related gene expression is a normal component of seedling development. Our results show that mitochondrial retrograde signaling represses the PCO N-degron pathway, enhancing substrate function, thus facilitating downstream stress responses. This work reveals a novel mechanism through which mitochondrial retrograde signaling influences nuclear response to hypoxia by inhibition of an ancient cytoplasmic pathway of eukaryotic oxygen sensing.

RESULTS AND DISCUSSION

The inner mitochondrial membrane protein UCP1 inhibits the PCO N-degron pathway

Mitochondrial genomic DNA codes for less than 1% of its ∼2,000 proteins; therefore, mitochondria rely on the nuclear genome to remotely regulate their function.[6] This nuclear-mitochondrion interaction requires inter-compartment signaling, which can be retrograde (mitochondria to nucleus) or anterograde (nucleus to mitochondria), acting to adjust organelle function during development and in response to environmental stresses.[7] Major advances have been made in both animals and plants in defining different molecular mechanisms regulating mitochondrial retrograde signaling.[6] Several extra-mitochondrial factors have been identified that play roles in retrograde and anterograde signaling for regulating the plant-specific ALTERNATIVE OXIDASE (AOX) and other mitochondrial proteins encoded by the nucleus. For example, it was shown in *Arabidopsis thaliana* that the endoplasmic reticulum-localized transcription factor ANAC017 is required for AOX1a activation.[8] Unknown signals (presumed to be reactive oxygen species, ROS[8]) from dysfunctional mitochondria lead to protease cleavage of ANAC017, releasing an amino-terminal (Nt−) fragment that relocates to the nucleus to activate expression of components of the mitochondrial dysfunction stimulon,[9] and ANAC017 was recently shown to be activated by submergence-associated oxidative stress[10]

This is an open access article under the CC BY license (http://creativecommons.org/licenses/by/4.0/).

and involved in submergence tolerance.[11] In diverse flowering plant taxa, UCP1 overexpression (*35S:UCP1ox*) decreases the release of ROS by mitochondria, protecting plants from multiple biotic and abiotic stresses,[4,12–14] and results in a broad alteration of the nuclear-derived transcriptome that interestingly includes activation of hypoxia (low oxygen)-responsive transcripts in *Nicotiana tabacum*.[3] Pharmacological induction of mitochondrial dysfunction was also shown to result in upregulation of hypoxia gene expression in several studies demonstrating similarities between inhibition of mitochondrial electron transport chain components and response to hypoxia.[15–17] These different datasets show that mitochondrial retrograde signaling enhances hypoxia-responsive gene expression in plants, though no mechanism has been described to account for this. As the mitochondrion is the major site of oxygen consumption in the cell, it might be expected that a relationship exists with cytoplasmic oxygen sensing, and links have been observed between the animal hypoxia-inducible factor (HIF) oxygen-sensing system and mitochondrial function.[18]

Plants have an oxygen-sensing mechanism that involves O_2-dependent destruction of protein substrates with amino-terminal Cys– through the PLANT CYSTEINE OXIDASE (PCO) branch of the PRT6 N-degron pathway (hereafter referred to as the PCO N-degron pathway), and major substrates of the pathway are the group VII ETHYLENE RESPONSE FACTOR (ERFVII) transcription factors[5] (Figure 1A). Under low-oxygen conditions, ERFVIIs are stabilized due to inhibition of PCO activity,[19,20] resulting in induction of hypoxia-related gene expression. We investigated whether the previously observed constitutive induction of hypoxia gene expression by UCP1 overexpression[3] was due to inhibition of PCO N-degron pathway activity in normoxia. An *A. thaliana* transgenic line, *35S:Met-Cys-^{HA}GUS*,[21] constitutively expressing an artificial PCO N-degron pathway substrate (that following Met removal by MetAP [Figure 1A] becomes a substrate due to N-terminal Cys) was used to analyze the effect of UCP1 on PCO N-degron pathway activity. We transformed this line with a construct harboring UCP1 under the control of the 35S promoter (*35S:UCP1*, hereafter UCP1ox) (Figure S1). Out of 15 transgenic events, three that presented distinct enhanced levels of UCP1 RNA and protein (Figures 1B and 1C) were selected for further analysis. The accumulation of ^{HA}GUS was significantly increased in all UCP1ox lines, observed both as increased histochemical staining in mature leaves and increased protein abundance via western blotting in both seedlings and mature leaves (Figures 1D and 1E).

We investigated if known inducers or inhibitors of UCP1 activity might affect the UCP1ox-induced stabilization of ^{HA}GUS using line E#4 (henceforth UCP1ox). A marked decrease in ^{HA}GUS protein was observed when leaf discs were treated with GTP (a classical UCP1 inhibitor[22]), while no influence was found for ADP treatment (a stimulator of coupled respiration not linked to UCP1 activity) (Figure 1F). UCP1 activator 4-hydroxynonenal (HNE)[14] increased ^{HA}GUS accumulation slightly compared to the ethanol control. We also tested if Asp and Glu, two amino acids proposed to be transported by UCP1,[2] influence PCO N-degron pathway activity and observed relatively less ^{HA}GUS protein in Asp-treated UCP1ox, compared to Glu treatment. It was previously shown that ethylene enhances *PHYTOGLOBIN* (*PGB*)1 expression, which in turn reduces NO levels and stabilizes ERFVIIs.[23] Interestingly, treatment with $AgNO_3$ (an inhibitor of ethylene perception) strongly reduced ^{HA}GUS accumulation, whereas ACC (an ethylene precursor and stimulator of ethylene synthesis) had no effect. To investigate whether inhibition of the PCO N-degron pathway may be a general action of mitochondrial retrograde signaling, we analyzed the influence of seedling treatment with salicylhydroxamic acid (SHAM) (a relatively selective *in vitro* competitive inhibitor of AOX). We observed increased stabilization of ^{HA}GUS in both wild type (WT) and UCP1ox, suggesting an additive effect on stabilization (Figure 1G). In addition, we observed increased accumulation of the ERFVII HRE2^{3xHA} (from the transgene *35S:HRE2^{3xHA}*[24]) when seedlings were treated with either SHAM or antimycin A (an inhibitor of cytochrome *c* reductase) (Figure S1C). These data suggest that alterations of the activities of discrete mitochondrial functions affect the activity of the cytoplasmic PCO N-degron pathway.

Overexpression of UCP1 enhances abiotic stress tolerance and hypoxia-related gene expression through ERFVII transcription factors

The PCO N-degron pathway has been shown to be a general sensor of plant stress.[21,25,26] The *prt6* mutant enhances tolerance to a number of abiotic and biotic stresses, whereas genetic removal of the *ERFVII* substrates of the PCO N-degron pathway increases susceptibility to those stresses.[21,23,24,26–31] We investigated whether genetic alteration of UCP1 activity (using UCP1ox and *ucp1* mutant) would alter the response to abiotic stresses. Similar to *prt6-1*, UCP1ox seedlings showed increased tolerance to high salt, and seedling root meristems to hypoxia, compared to WT Col-0 accession, and root meristems of *ucp1* were more sensitive to hypoxia stress (Figures 2A and 2B). At a later stage of development (starting at 21 days old), UCP1ox irrigated for 3 weeks with 150 mM NaCl exhibited enhanced salt tolerance compared to WT and *ucp1*. Conductivity, an indirect measurement of ion leakage, increased 7-fold in WT plants after 1 week irrigation with high salt compared to 4- and 5-fold increase in UCP1ox and *prt6-1*, respectively (Figures 2C and 2D). As hypoxia gene expression is increased in response to ERFVII stabilization in *A. thaliana*[32,33] and UCP1 overexpression in *N. tabacum*,[3] we analyzed, in *A. thaliana*, the influence of UCP1 on the expression of selected members of the "core 49" genes with conserved hypoxia induction:[34] *ALCOHOL DEHYDROGENASE* (*ADH*)*1*; *PGB1*, recently shown to prime ethylene-mediated pre-adaptation to hypoxia;[23] and *CALMODULIN-LIKE* (*CML*)*38*, a calcium ion sensor localizing to ribo-nucleoprotein complexes in hypoxia.[35] In both seedlings and mature leaves, UCP1ox greatly increased transcript accumulation of the target genes, whereas this was strongly reduced in *ucp1* in comparison to WT for all three genes (Figure 2E). As expected, expression was increased in *prt6*, at a similar level to *prt6 ucp1*, which indicates that *PRT6* acts downstream of *UCP1* in controlling hypoxia gene expression (because lack of *PRT6* constitutively stabilizes ERFVIIs). Because *UCP1* was previously shown to enhance AOX1 protein accumulation,[12,36] we analyzed *UCP1* influence on the expression of other mitochondrially located proteins encoded by nuclear genes. Interestingly, in both seedlings and mature leaves, expression of *NAD(P)H DEHYDROGENASE B4* (*NDB4*) (previously shown to be a marker for mitochondrial retrograde signaling[37]) was induced in UCP1ox, *prt6*, and *prt6 ucp1* but

CellPress
OPEN ACCESS

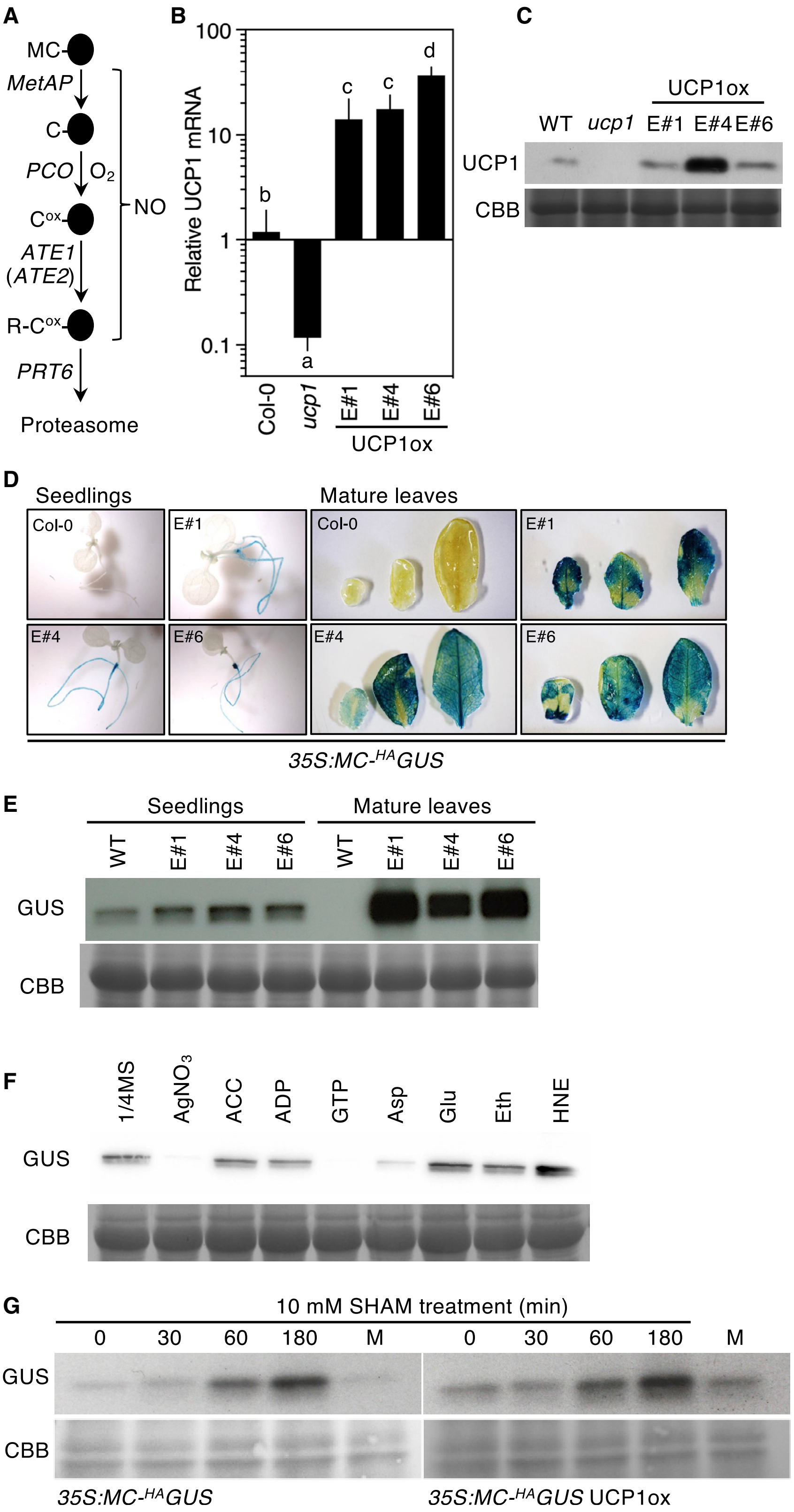

Figure 1. Genetic alteration of mitochondrial UCP1 abundance influences the stability of artificial PCO N-degron pathway substrate Cys-^{HA}GUS

(A) Schematic representation of the PLANT CYSTEINE OXIDASE (PCO) N-degron pathway. Black ovals indicate proteins, amino terminal (Nt) amino acids are single letter codes, and ox indicates oxidized cysteine. MetAP, METHIONINE AMINO-PEPTIDASE; ATE, ARGINYL TRANSFERASE; PRT6, PROTEOLYSIS6; O_2, oxygen; NO, nitric oxide.

(B and C) UCP1 RNA (B) and protein expression (C) compared to Col-0 *35S:Met-Cys-^{HA}GUS* (WT) in three independent transgenic lines containing *35S:UCP1* (E#1, #4, and #6) and the *ucp1* mutant.

(D and E) Histochemical visualization of GUS activity (D) and western blot analysis (E) of ^{HA}GUS protein abundance in whole seedlings or mature leaves.

(F) Western blot of ^{HA}GUS in UCP1ox leaf discs incubated for 30 min in the dark in ½ MS medium supplemented or not with $AgNO_3$ (100 μM), ACC (10 μM), ADP (100 μM), GTP (100 μM), aspartate (5 mM), glutamate (5 mM), ethanol (Eth) (0.1%), or HNE (in 0.1% ethanol) (30 μM).

(G) Western blot analysis of ^{HA}GUS abundance following treatment with SHAM (10 mM). M indicates mock samples sprayed with 2% ethanol only. CBB, Coomassie Brilliant Blue loading control.

Error bars indicate SD; letters one-way ANOVA. See also Figure S1.

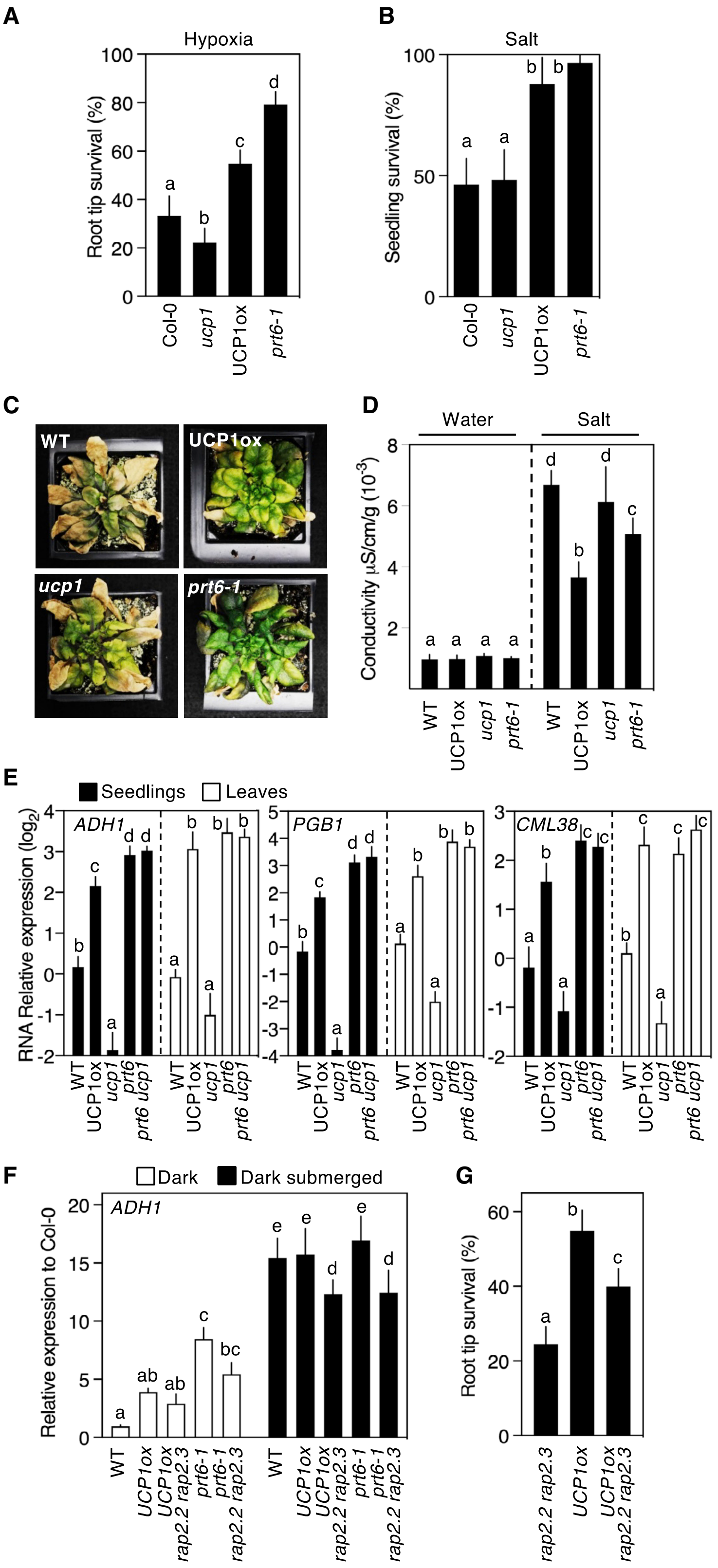

Figure 2. Stress tolerance and gene expression are enhanced in UCP1ox plants through ERFVII substrates of the PCO N-degron pathway

(A) Root tip survival in response to hypoxia.
(B) Seedling survival following growth on media containing NaCl (125 mM).
(C) Images of plants irrigated with 150 mM NaCl for 3 weeks.
(D) Conductivity (electrolyte leakage) of mature leaves collected from plants watered with 150 mM NaCl or water for 1 week.
(E) Relative expression of transcripts for *ADH1*, *PGB1*, and *CML38* in 7-day-old seedlings and mature leaves.
(F) Relative *ADH1* transcript levels in control (1 h dark) and treated (1 h submerged+dark) seedlings for WT, mutants, and UCP1ox combinations.
(G) Root tip survival in response to hypoxia (Col-0 shown in A).
Error bars indicate SD; letters one-way ANOVA. See also Figure S2.

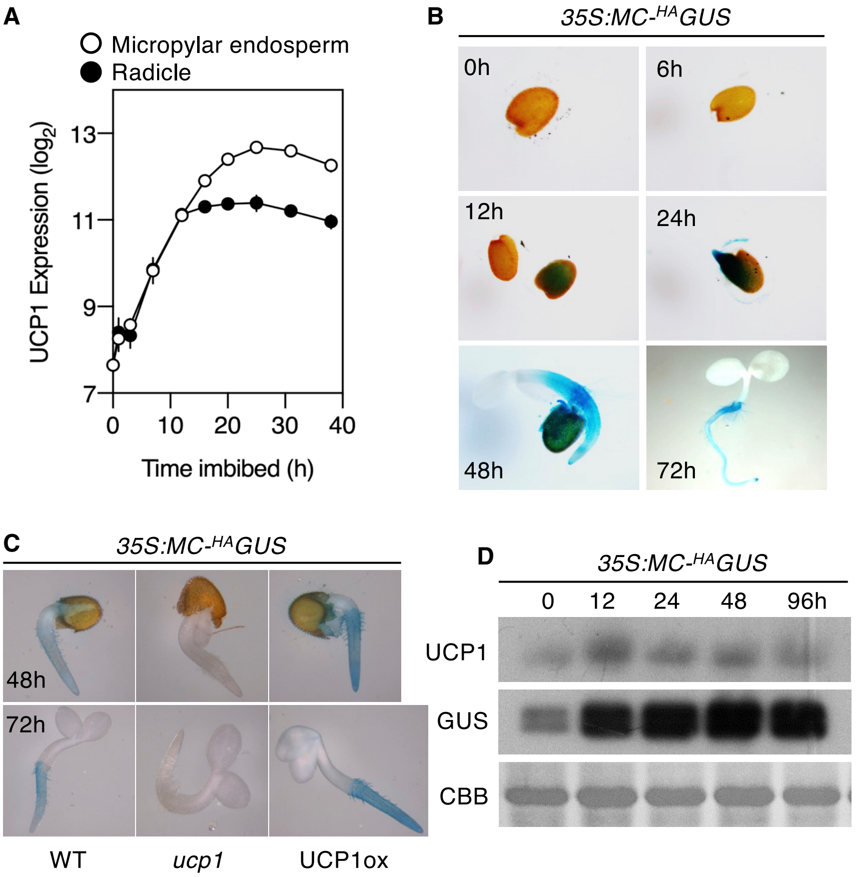

Figure 3. Expression of *UCP1* during germination influences GUS accumulation

(A) Expression of *UCP1* during germination and seedling establishment.[41]

(B) Histochemical staining of *35S:MC-^{HA}GUS* (hours imbibition in the light).

(C) Comparison of histochemical staining for *35S:MC-^{HA}GUS* in WT, *ucp1*, and UCP1ox backgrounds (hours imbibition in the light).

(D) Western blot analysis of ^{HA}GUS and UCP1 in mature leaves following transfer to 4°C in the light (h).

See also Figure S3.

reduced in *erfVII* and *ucp1* compared to Col-0 (Figure S2A). A similar expression profile was also observed for the nuclear-encoded mitochondrial inner membrane protein *DICARBOXYLATE CARRIER* (*DIC*)*3* (important for the redox connection between mitochondria and cytoplasm[38]).

To demonstrate a direct link between UCP1 and potential downstream ERFVII activities, we analyzed gene expression and physiological tolerance to hypoxia in UCP1ox *rap2.2 rap2.3*, a combination that removes two of the three constitutively highly expressed (at the RNA level) ERFVIIs. After 1 h submergence in the dark (to simulate flooding stress), the expression of *ADH1*, *PGB1*, and *CML38* was enhanced in all lines tested, but a significant reduced induction was observed in both UCP1ox *rap2.2 rap2.3* versus UCP1ox and prt6-*1 rap2.2 rap2.3* versus *prt6-1* (Figures 2F and S2B). Hypoxia tolerance, measured as seedling root meristem survival,[23] was also reduced in UCP1ox *rap2.2 rap2.3* compared to UCP1ox, though was still greater than *rap2.2 rap2.3* (Figure 2G). Taken together with the observation of the inhibition of the PCO N-degron pathway by UCP1 (Figure 1), these data indicate that UCP1 acts through ERFVIIs in regulating hypoxia gene expression and tolerance to hypoxia and salt stress.

UCP1 inhibition of the PCO/PRT6 N-degron pathway is a normal part of seedling development

Transcripts of *UCP1* are ubiquitously expressed across *A. thaliana* tissues but are upregulated under particular developmental conditions, particularly during germination (Figure S3A). We hypothesized that PCO N-degron pathway substrates may be stabilized under physiological and tissue/temporal conditions where *UCP1* is relatively highly expressed. RNA expression of *UCP1* increases strongly during seedling germination and establishment (Figure 3A; see imbibed seeds 24 h; Figure S3A), a feature that was strongly correlated with an increase in GUS activity in *35S:Met-Cys-^{HA}GUS* seedlings (Figure 3B). To determine if this pattern of ^{HA}GUS stabilization was a consequence of *UCP1*, we crossed *35S:Met-Cys-^{HA}GUS* to a *ucp1* mutant with significantly reduced levels of *UCP1* expression (Figure 1B). Remarkably, the *ucp1* mutation abolished ^{HA}GUS accumulation in seedlings, despite equivalent levels of transgene expression (Figures 3C and S3B). Treatment with the proteasome inhibitor bortezomib increased ^{HA}GUS accumulation in the roots of *ucp1*, indicating that its absence in this background was due to elevated proteasomal degradation relative to WT. (Figure S3B). In addition, a correlation between increased *UCP1* RNA and protein (as reported previously in several species[39]) and ^{HA}GUS protein accumulation was observed when mature plants were subjected to cold stress (Figures 3D and S3C). Cold-induced UCP1 expression may represent a mechanism for enhancing PCO N-degron pathway substrate accumulation in response to low temperatures, which we previously reported for the ERFVIIs and VERNALIZATION2.[25] We also investigated whether UCP1 expression is altered during submergence, a condition that stabilizes the substrates of the PCO N-degron pathway due to reduced oxygen,[24,40] and found that UCP1 protein accumulated during submergence in the dark whereas it decreased when plants were maintained only in the dark (Figure S3D). ^{HA}GUS was strongly stabilized under submergence in the dark after only 10 min.

To analyze the role of *UCP1* in influencing gene expression during germination, we examined differential RNA expression between WT (Col-0) and *ucp1* for known hypoxia-related genes comparing pre-chilled seeds following transfer to the light for 24, 48, and 72 h (Figure 4A). Interestingly, the transcripts for several genes, including *ADH1*, *PGB1*, *ACO1*, and *PDC2*, showed a similar lower expression pattern in *ucp1* compared to WT at

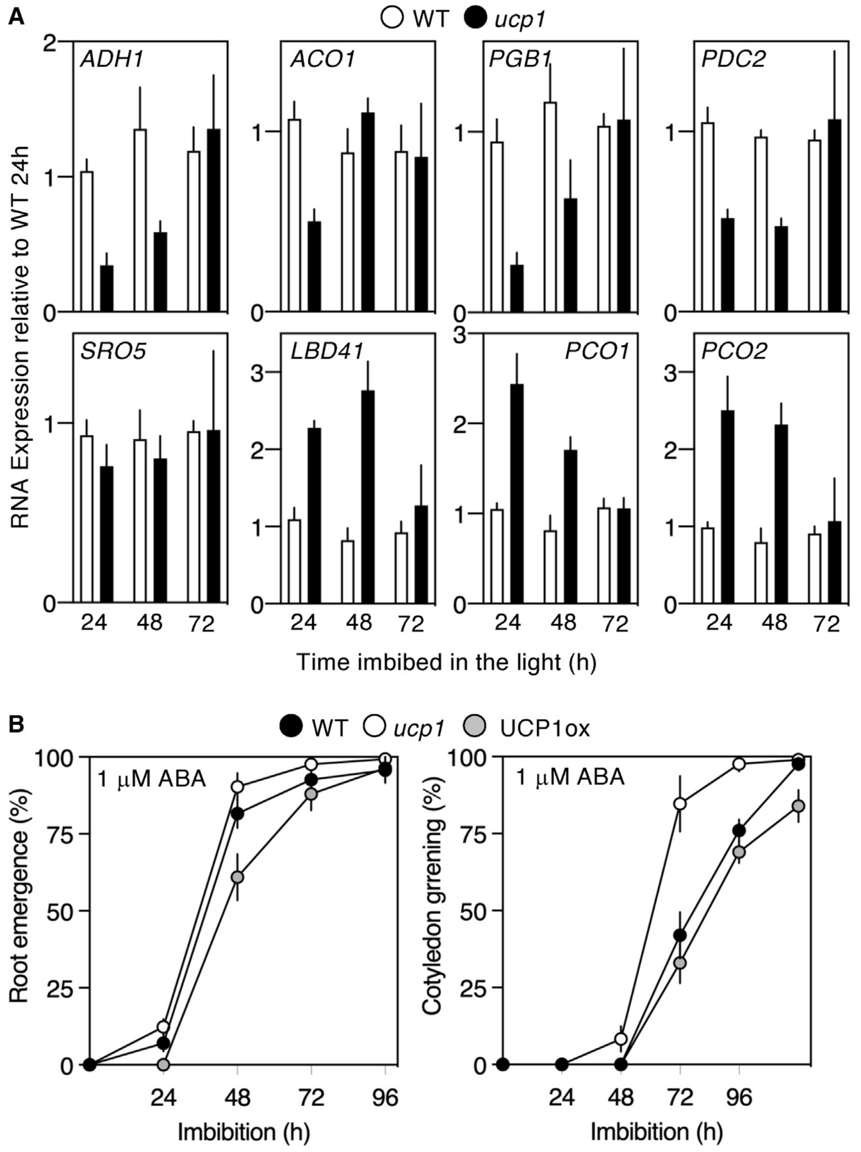

Figure 4. Influence of *UCP1* on gene expression and physiology of germination

(A) Relative expression of hypoxia-related transcripts in WT and *ucp1* during germination.

(B) Germination (root emergence) and establishment (cotyledon greening) of WT, *ucp1*, and UCP1ox on media containing 1 μM ABA.

Error bars indicate SD. See also Figure S4.

the beginning of the time course, increasing toward the end. Other hypoxia-related transcripts[34] either showed no difference in expression or increased expression (e.g., *PCO1* and *2*). These data indicate that *UCP1* is required for expression of some, but not all, hypoxia-related genes during early germination. The importance of fermentative metabolism, in particular of ADH1, was recently demonstrated for plants growing under normal non-hypoxic conditions,[42] and increased levels of *PGB1* were shown to reduce NO levels and enhance stabilization of Cys-2 N-degron substrates.[23] Increased hypoxia-related gene expression in seedlings of Col-0 and *ucp1* following submergence was similar, indicating that inhibition of the PCO N-degron pathway by hypoxia is downstream of *UCP1* function (Figure S4A) because reduced environmental oxygen levels stabilize pathway substrates due to inhibition of PCO activity,[19,20] regardless of upstream regulation. This requirement may contribute to ecologically relevant abiotic stress tolerance during this early developmental period.[43] In support of this hypothesis, the *ucp1* mutant exhibited more rapid establishment than WT on media plates containing abscisic acid (1 μM ABA), resembling responses observed in ERFVII mutants,[21,28] while in the UCP1ox line establishment was delayed, and no differences in germination were observed on media without ABA (Figures 4B and S4B).

It has previously been shown that chemically or genetically induced mitochondrial dysfunction increased hypoxia-associated gene expression, suggesting the existence of a novel pathway of plant mitochondrial retrograde signaling.[17] In the present work we describe a mechanism linking the activity of mitochondrial inner-membrane protein UCP1 to inhibition of the PCO N-degron pathway, which controls gene expression under hypoxia and abiotic stress tolerance, and show that this is a normal feature of plant development. Our observations that chemical treatment with SHAM and antimycin A also inhibit the PCO N-degron pathway are indicative of a more general mechanism of mitochondrial retrograde signaling. It was previously shown that AOX protein is reduced in the *ucp1* mutant[36] and increased in UCP1ox tobacco leaves,[3] highlighting possible interactions between different pathways. In addition to this, we observed a similar UCP1-regulated expression of transcripts for nuclear-encoded mitochondrial marker proteins that was regulated through PRT6. The PRT6/UBR N-degron pathway evolved early in eukaryotes,[44] and the fact that mammals possess a PCO counterpart, ADO,[45] raises the possibility that modulation of the animal ADO N-degron pathway could also act as part of a mechanism of mitochondrial retrograde signaling. The regulation of hypoxia responses by UCPs is not restricted to plants; for example, the lack of UCP1 in mouse brown adipose tissue is sufficient to decrease cold-inducible hypoxia through downregulation of hypoxia-inducible factor 1A (HIF-1A).[46,47] Likewise, the activity of human UCP2 is strongly associated with HIF-1A stabilization in distinct cancer cells.[48] It has been described recently that during germination, seed response to mitochondrial ROS production and, subsequently, ethylene induces a mitochondrial retrograde response.[49] Our results build on that report as we show that during germination UCP1 regulates PCO N-degron pathway function.

It remains unknown how UCP1 influences mitochondrial signaling, especially because of the multiple functions that have been assigned to the protein.[1,2] It is possible that it can work as an uncoupler that limits ROS production by preventing excessive reduction levels of electron transport components, in addition to a recently identified function as a transporter. How the mitochondrial activity of UCP1 leads to cytoplasmic stabilization of Cys-2 N-degron substrates is also unclear. Although signals for chloroplast retrograde signaling are known,[6] none so far have been identified for plant mitochondrial retrograde signaling. Of the known requirements for activity of the PCO N-degron pathway (Figure 1A),[5] it is unlikely that UCP1 activity influences cellular oxygen levels. Moreover, NO levels increase in UCP1ox lines, rather than decrease, which would be required to inhibit the pathway[21,28] (Figure S4C). Comparison of mitochondrially induced cytoplasmic changes with factors influencing the kinetic properties of enzymes in the PCO N-degron pathway may help to focus identification of important features.[17,19] Interestingly, *PCO1* and *PCO2* transcripts were upregulated in the *ucp1* mutant and downregulated in UCP1ox (Figure S4D), suggesting that UCP1-mediated changed PCO activity may result in altered pathway activity, resulting in the observed changed stability of ^{HA}GUS.

A mechanism of retrograde signaling linking ANAC017 with submergence has been described,[10,11] and although different from the mechanism described here, it will be important to understand its possible crosstalk with UCP1-mediated signaling that functions during normoxia. Together with the already known roles of the PCO N-degron pathway in oxygen and NO sensing, this work shows that the pathway forms part of a mitochondrial retrograde signaling mechanism linking oxygen consumption (in the mitochondrion) with oxygen sensing (in the cytoplasm) with importance for both normal development and responses to environmental stress.

STAR★METHODS

Detailed methods are provided in the online version of this paper and include the following:

- KEY RESOURCES TABLE
- RESOURCE AVAILABILITY
  - Lead contact
  - Materials availability
  - Data and code availability
- EXPERIMENTAL MODEL AND SUBJECT DETAILS
- METHOD DETAILS
  - Plant transformation
  - Analysis of plant growth and response to stresses
  - Gene expression analyses
- QUANTIFICATION AND STATISTICAL ANALYSIS

SUPPLEMENTAL INFORMATION

Supplemental information can be found online at https://doi.org/10.1016/j.cub.2022.01.037.

ACKNOWLEDGMENTS

P.B. was a FAPESP (2014/17634-5, 2015/24881-1, and 2017/22745-9) and CAPES (88887.572598/2020-00) postdoc research fellow. This work was supported by the Biotechnology and Biological Sciences Research Council (grant nos. BB/R002428/1 and BB/S005293/1) to M.J.H. P.A. was supported by the FAPESP grant 2016/23218-0 through the Genomics for Climate Change Research Center (GCCRC). I.G.M. is a CNPq productivity research fellow. D.J.G. and R.O. were supported by the European Research Council (ERC Starting Grant 715441-GasPlaNt).

AUTHOR CONTRIBUTIONS

P.B., M.J.H., and P.A. conceived the project and designed experiments. P.B., C.D., R.O., G.S., J.V., and M.J.H. performed the experiments. P.B., C.D., J.Y., D.J.G., I.G.M., M.J.H., and P.A. analyzed the data. M.J.H., P.B., and P.A. wrote the manuscript.

DECLARATION OF INTERESTS

The authors declare no competing interests.

Received: November 1, 2021
Revised: December 10, 2021
Accepted: January 12, 2022
Published: February 2, 2022

REFERENCES

1. Ježek, P., Holendová, B., Garlid, K.D., and Jabůrek, M. (2018). Mitochondrial uncoupling proteins: subtle regulators of cellular redox signaling. Antioxid. Redox Signal. *29*, 667–714.
2. Monné, M., Daddabbo, L., Gagneul, D., Obata, T., Hielscher, B., Palmieri, L., Miniero, D.V., Fernie, A.R., Weber, A.P.M., and Palmieri, F. (2018). Uncoupling proteins 1 and 2 (UCP1 and UCP2) from *Arabidopsis thaliana* are mitochondrial transporters of aspartate, glutamate, and dicarboxylates. J. Biol. Chem. *293*, 4213–4227.
3. Barreto, P., Okura, V., Pena, I.A., Maia, R., Maia, I.G., and Arruda, P. (2016). Overexpression of mitochondrial uncoupling protein 1 (UCP1) induces a hypoxic response in *Nicotiana tabacum* leaves. J. Exp. Bot. *67*, 301–313.
4. Barreto, P., Yassitepe, J.E.C.T., Wilson, Z.A., and Arruda, P. (2017). Mitochondrial uncoupling protein 1 overexpression increases yield in *Nicotiana tabacum* under drought stress by improving source and sink metabolism. Front. Plant Sci. *8*, 1836.
5. Holdsworth, M.J., Vicente, J., Sharma, G., Abbas, M., and Zubrycka, A. (2020). The plant N-degron pathways of ubiquitin-mediated proteolysis. J. Integr. Plant Biol. *62*, 70–89.
6. Pfannschmidt, T., Terry, M.J., Van Aken, O., and Quiros, P.M. (2020). Retrograde signals from endosymbiotic organelles: a common control principle in eukaryotic cells. Philos. Trans. R. Soc. Lond. B Biol. Sci. *375*, 20190396.
7. Crawford, T., Lehotai, N., and Strand, Å. (2018). The role of retrograde signals during plant stress responses. J. Exp. Bot. *69*, 2783–2795.
8. Ng, S., Ivanova, A., Duncan, O., Law, S.R., Van Aken, O., De Clercq, I., Wang, Y., Carrie, C., Xu, L., Kmiec, B., et al. (2013). A membrane-bound NAC transcription factor, ANAC017, mediates mitochondrial retrograde signaling in Arabidopsis. Plant Cell *25*, 3450–3471.
9. Meng, X., Li, L., De Clercq, I., Narsai, R., Xu, Y., Hartmann, A., Claros, D.L., Custovic, E., Lewsey, M.G., Whelan, J., and Berkowitz, O. (2019). ANAC017 coordinates organellar functions and stress responses by reprogramming retrograde signaling. Plant Physiol. *180*, 634–653.
10. Bui, L.T., Shukla, V., Giorgi, F.M., Trivellini, A., Perata, P., Licausi, F., and Giuntoli, B. (2020). Differential submergence tolerance between juvenile and adult Arabidopsis plants involves the ANAC017 transcription factor. Plant J. *104*, 979–994.
11. Meng, X., Li, L., Narsai, R., De Clercq, I., Whelan, J., and Berkowitz, O. (2020). Mitochondrial signalling is critical for acclimation and adaptation to flooding in *Arabidopsis thaliana*. Plant J. *103*, 227–247.

12. Barreto, P., Okura, V.K., Neshich, I.A.P., Maia, Ide.G., and Arruda, P. (2014). Overexpression of UCP1 in tobacco induces mitochondrial biogenesis and amplifies a broad stress response. BMC Plant Biol. *14*, 144.

13. Chen, S., Liu, A., Zhang, S., Li, C., Chang, R., Liu, D., Ahammed, G.J., and Lin, X. (2013). Overexpression of mitochondrial uncoupling protein conferred resistance to heat stress and *Botrytis cinerea* infection in tomato. Plant Physiol. Biochem. *73*, 245–253.

14. Smith, A.M.O., Ratcliffe, R.G., and Sweetlove, L.J. (2004). Activation and function of mitochondrial uncoupling protein in plants. J. Biol. Chem. *279*, 51944–51952.

15. Schmidt, R.R., Fulda, M., Paul, M.V., Anders, M., Plum, F., Weits, D.A., Kosmacz, M., Larson, T.R., Graham, I.A., Beemster, G.T.S., et al. (2018). Low-oxygen response is triggered by an ATP-dependent shift in oleoyl-CoA in *Arabidopsis*. Proc. Natl. Acad. Sci. USA *115*, E12101–E12110.

16. Wagner, S., Steinbeck, J., Fuchs, P., Lichtenauer, S., Elsässer, M., Schippers, J.H.M., Nietzel, T., Ruberti, C., Van Aken, O., Meyer, A.J., et al. (2019). Multiparametric real-time sensing of cytosolic physiology links hypoxia responses to mitochondrial electron transport. New Phytol. *224*, 1668–1684.

17. Wagner, S., Van Aken, O., Elsässer, M., and Schwarzländer, M. (2018). Mitochondrial energy signaling and its role in the low-oxygen stress response of plants. Plant Physiol. *176*, 1156–1170.

18. Thomas, L.W., and Ashcroft, M. (2019). Exploring the molecular interface between hypoxia-inducible factor signalling and mitochondria. Cell. Mol. Life Sci. *76*, 1759–1777.

19. White, M.D., Kamps, J.J.A.G., East, S., Taylor Kearney, L.J., and Flashman, E. (2018). The plant cysteine oxidases from *Arabidopsis thaliana* are kinetically tailored to act as oxygen sensors. J. Biol. Chem. *293*, 11786–11795.

20. White, M.D., Klecker, M., Hopkinson, R.J., Weits, D.A., Mueller, C., Naumann, C., O'Neill, R., Wickens, J., Yang, J., Brooks-Bartlett, J.C., et al. (2017). Plant cysteine oxidases are dioxygenases that directly enable arginyl transferase-catalysed arginylation of N-end rule targets. Nat. Commun. *8*, 14690.

21. Vicente, J., Mendiondo, G.M., Movahedi, M., Peirats-Llobet, M., Juan, Y.T., Shen, Y.Y., Dambire, C., Smart, K., Rodriguez, P.L., Charng, Y.Y., et al. (2017). The Cys-Arg/N-end rule pathway is a general sensor of abiotic stress in flowering plants. Curr. Biol. *27*, 3183–3190.e4.

22. Vercesi, A.E., Borecký, J., Maia, Ide.G., Arruda, P., Cuccovia, I.M., and Chaimovich, H. (2006). Plant uncoupling mitochondrial proteins. Annu. Rev. Plant Biol. *57*, 383–404.

23. Hartman, S., Liu, Z., van Veen, H., Vicente, J., Reinen, E., Martopawiro, S., Zhang, H., van Dongen, N., Bosman, F., Bassel, G.W., et al. (2019). Ethylene-mediated nitric oxide depletion pre-adapts plants to hypoxia stress. Nat. Commun. *10*, 4020.

24. Gibbs, D.J., Lee, S.C., Isa, N.M., Gramuglia, S., Fukao, T., Bassel, G.W., Correia, C.S., Corbineau, F., Theodoulou, F.L., Bailey-Serres, J., and Holdsworth, M.J. (2011). Homeostatic response to hypoxia is regulated by the N-end rule pathway in plants. Nature *479*, 415–418.

25. Gibbs, D.J., Tedds, H.M., Labandera, A.M., Bailey, M., White, M.D., Hartman, S., Sprigg, C., Mogg, S.L., Osborne, R., Dambire, C., et al. (2018). Oxygen-dependent proteolysis regulates the stability of angiosperm polycomb repressive complex 2 subunit VERNALIZATION 2. Nat. Commun. *9*, 5438.

26. Vicente, J., Mendiondo, G.M., Pauwels, J., Pastor, V., Izquierdo, Y., Naumann, C., Movahedi, M., Rooney, D., Gibbs, D.J., Smart, K., et al. (2019). Distinct branches of the N-end rule pathway modulate the plant immune response. New Phytol. *221*, 988–1000.

27. Abbas, M., Berckhan, S., Rooney, D.J., Gibbs, D.J., Vicente Conde, J., Sousa Correia, C., Bassel, G.W., Marín-de la Rosa, N., León, J., Alabadí, D., et al. (2015). Oxygen sensing coordinates photomorphogenesis to facilitate seedling survival. Curr. Biol. *25*, 1483–1488.

28. Gibbs, D.J., Md Isa, N., Movahedi, M., Lozano-Juste, J., Mendiondo, G.M., Berckhan, S., Marín-de la Rosa, N., Vicente Conde, J., Sousa Correia, C., Pearce, S.P., et al. (2014). Nitric oxide sensing in plants is mediated by proteolytic control of group VII ERF transcription factors. Mol. Cell *53*, 369–379.

29. Gravot, A., Richard, G., Lime, T., Lemarié, S., Jubault, M., Lariagon, C., Lemoine, J., Vicente, J., Robert-Seilaniantz, A., Holdsworth, M.J., and Manzanares-Dauleux, M.J. (2016). Hypoxia response in Arabidopsis roots infected by *Plasmodiophora brassicae* supports the development of clubroot. BMC Plant Biol. *16*, 251.

30. Holman, T.J., Jones, P.D., Russell, L., Medhurst, A., Ubeda Tomás, S., Talloji, P., Marquez, J., Schmuths, H., Tung, S.A., Taylor, I., et al. (2009). The N-end rule pathway promotes seed germination and establishment through removal of ABA sensitivity in Arabidopsis. Proc. Natl. Acad. Sci. USA *106*, 4549–4554.

31. Mendiondo, G.M., Gibbs, D.J., Szurman-Zubrzycka, M., Korn, A., Marquez, J., Szarejko, I., Maluszynski, M., King, J., Axcell, B., Smart, K., et al. (2016). Enhanced waterlogging tolerance in barley by manipulation of expression of the N-end rule pathway E3 ligase PROTEOLYSIS6. Plant Biotechnol. J. *14*, 40–50.

32. Bailey-Serres, J., Fukao, T., Gibbs, D.J., Holdsworth, M.J., Lee, S.C., Licausi, F., Perata, P., Voesenek, L.A., and van Dongen, J.T. (2012). Making sense of low oxygen sensing. Trends Plant Sci. *17*, 129–138.

33. van Dongen, J.T., and Licausi, F. (2015). Oxygen sensing and signaling. Annu. Rev. Plant Biol. *66*, 345–367.

34. Mustroph, A., Lee, S.C., Oosumi, T., Zanetti, M.E., Yang, H., Ma, K., Yaghoubi-Masihi, A., Fukao, T., and Bailey-Serres, J. (2010). Cross-kingdom comparison of transcriptomic adjustments to low-oxygen stress highlights conserved and plant-specific responses. Plant Physiol. *152*, 1484–1500.

35. Lokdarshi, A., Conner, W.C., McClintock, C., Li, T., and Roberts, D.M. (2016). Arabidopsis CML38, a calcium sensor that localizes to ribonucleoprotein complexes under hypoxia stress. Plant Physiol. *170*, 1046–1059.

36. Sweetlove, L.J., Lytovchenko, A., Morgan, M., Nunes-Nesi, A., Taylor, N.L., Baxter, C.J., Eickmeier, I., and Fernie, A.R. (2006). Mitochondrial uncoupling protein is required for efficient photosynthesis. Proc. Natl. Acad. Sci. USA *103*, 19587–19592.

37. De Clercq, I., Vermeirssen, V., Van Aken, O., Vandepoele, K., Murcha, M.W., Law, S.R., Inzé, A., Ng, S., Ivanova, A., Rombaut, D., et al. (2013). The membrane-bound NAC transcription factor ANAC013 functions in mitochondrial retrograde regulation of the oxidative stress response in Arabidopsis. Plant Cell *25*, 3472–3490.

38. Palmieri, L., Picault, N., Arrigoni, R., Besin, E., Palmieri, F., and Hodges, M. (2008). Molecular identification of three *Arabidopsis thaliana* mitochondrial dicarboxylate carrier isoforms: organ distribution, bacterial expression, reconstitution into liposomes and functional characterization. Biochem. J. *410*, 621–629.

39. Barreto, P., Couñago, R.M., and Arruda, P. (2020). Mitochondrial uncoupling protein-dependent signaling in plant bioenergetics and stress response. Mitochondrion *53*, 109–120.

40. Licausi, F., Kosmacz, M., Weits, D.A., Giuntoli, B., Giorgi, F.M., Voesenek, L.A.C.J., Perata, P., and van Dongen, J.T. (2011). Oxygen sensing in plants is mediated by an N-end rule pathway for protein destabilization. Nature *479*, 419–422.

41. Dekkers, B.J.W., Pearce, S., van Bolderen-Veldkamp, R.P., Marshall, A., Widera, P., Gilbert, J., Drost, H.G., Bassel, G.W., Müller, K., King, J.R., et al. (2013). Transcriptional dynamics of two seed compartments with opposing roles in Arabidopsis seed germination. Plant Physiol. *163*, 205–215.

42. Ventura, I., Brunello, L., Iacopino, S., Valeri, M.C., Novi, G., Dornbusch, T., Perata, P., and Loreti, E. (2020). Arabidopsis phenotyping reveals the importance of alcohol dehydrogenase and pyruvate decarboxylase for aerobic plant growth. Sci. Rep. *10*, 16669.

CellPress
OPEN ACCESS

43. Green, P.T., Harms, K.E., and Connell, J.H. (2014). Nonrandom, diversifying processes are disproportionately strong in the smallest size classes of a tropical forest. Proc. Natl. Acad. Sci. USA *111*, 18649–18654.

44. Holdsworth, M.J., and Gibbs, D.J. (2020). Comparative biology of oxygen sensing in plants and animals. Curr. Biol. *30*, R362–R369.

45. Masson, N., Keeley, T.P., Giuntoli, B., White, M.D., Puerta, M.L., Perata, P., Hopkinson, R.J., Flashman, E., Licausi, F., and Ratcliffe, P.J. (2019). Conserved N-terminal cysteine dioxygenases transduce responses to hypoxia in animals and plants. Science *365*, 65–69.

46. Xue, Y., Petrovic, N., Cao, R., Larsson, O., Lim, S., Chen, S., Feldmann, H.M., Liang, Z., Zhu, Z., Nedergaard, J., et al. (2009). Hypoxia-independent angiogenesis in adipose tissues during cold acclimation. Cell Metab. *9*, 99–109.

47. Basse, A.L., Isidor, M.S., Winther, S., Skjoldborg, N.B., Murholm, M., Andersen, E.S., Pedersen, S.B., Wolfrum, C., Quistorff, B., and Hansen, J.B. (2017). Regulation of glycolysis in brown adipocytes by HIF-1α. Sci. Rep. *7*, 4052.

48. Baffy, G. (2017). Mitochondrial uncoupling in cancer cells: liabilities and opportunities. Biochim. Biophys. Acta. Bioenerg. *1858*, 655–664.

49. Jurdak, R., Launay-Avon, A., Paysant-Le Roux, C., and Bailly, C. (2021). Retrograde signalling from the mitochondria to the nucleus translates the positive effect of ethylene on dormancy breaking of Arabidopsis thaliana seeds. New Phytol. *229*, 2192–2205.

50. Nakagawa, T., Kurose, T., Hino, T., Tanaka, K., Kawamukai, M., Niwa, Y., et al. (2007). Development of series of gateway binary vectors, pGWBs, for realizing efficient construction of fusion genes for plant transformation. J. Biosci. Bioeng. *104*, 34–41.

51. Clough, S.J., and Bent, A.F. (1998). Floral dip: a simplified method for *Agrobacterium*-mediated transformation of *Arabidopsis thaliana*. Plant J. *16*, 735–743.

52. Dietrich, D., Schmuths, H., De Marcos Lousa, C., Baldwin, J.M., Baldwin, S.A., Baker, A., Theodoulou, F.L., and Holdsworth, M.J. (2009). Mutations in the Arabidopsis peroxisomal ABC transporter COMATOSE allow differentiation between multiple functions in planta: insights from an allelic series. Mol. Biol. Cell *20*, 530–543.

53. Melotto, M., Underwood, W., Koczan, J., Nomura, K., and He, S.Y. (2006). Plant stomata function in innate immunity against bacterial invasion. Cell *126*, 969–980.

STAR★METHODS

KEY RESOURCES TABLE

REAGENT or RESOURCE	SOURCE	IDENTIFIER
**Antibodies**		
Monoclonal Anti-HA antibody produced in mouse	Sigma-Aldrich	H3663; RRID:AB_262051
Goat anti-Mouse IgG1 Secondary Antibody	ThermoFisher	PA1-74421; RRID:AB_10988195
Anti-UCP - uncoupling protein Antibody	Agrisera	AS12 1850; RRID:AB_2904553
Secondary Antibody: (goat) anti-rabbit IgG HRP conjugate	Invitrogen	G21234; RRID:AB_2536530
Antibody detection kit: Pierce ECL Western Blotting Substrate	ThermoFisher	32106
Anti-β-Glucuronidase Antibody	Sigma-Aldrich	G5420; RRID:AB_477020
Anti-Rabbit-HRP conjugate	Sigma-Aldrich	A0545; RRID:AB_257896
**Chemicals, peptides, and recombinant proteins**		
Murashige and Skoog (MS) medium	Sigma-Aldrich	M5524
(±)-Abscisic acid	Sigma-Aldrich	A1049
DAF-2 DA (4,5-diaminofluorescein diacetate)	Sigma-Aldrich	D2813-1MG
X-Gluc solution (5-bromo-4-chloro-3-indolyl-beta-Dglucuronic acid, cyclohexylammonium salt)	X-GLUC Direct	X-Gluc
4-hydroxy Nonenal (HNE)	Cayman	32100
L-Aspartic acid	Sigma-Aldrich	A9256
L-Glutamic acid	Sigma-Aldrich	G1251
Salicylhydroxamic acid (SHAM)	Sigma-Aldrich	S607
DAF-2 DA (4,5-diaminofluorescein diacetate)	Sigma-Aldrich	D2813-1MG
**Experimental models: Organisms/strains**		
Arabidopsis thaliana Col-0	NASC	N1092
Arabidopsis thaliana ucp1	[36]	NASC: N811222, SAIL242_A09C
Arabidopsis thaliana 35S:UCP1 in *35S:Met-Cys-HAGUS*	This study	N/A
Arabidopsis thaliana ucp1 in *35S:Met-Cys-HAGUS*	This study	N/A
Arabidopsis thaliana rap2.2 rap2.3, prt6 rap2.2 rap2.3	[28]	*rap2.2 rap2.3, prt6 rap2.2 rap2.3*
Arabidopsis thaliana 35S:UCP1 in *rap2.2 rap2.3*	This study	35S:UCP1 in *rap2.2 rap2.3*
Arabidopsis thaliana prt6-1	[30]	NASC: N9873, SAIL 1278_H11
Arabidopsis thaliana ucp1 prt6-1	This study	*ucp1 prt6*
Arabidopsis thaliana 35S:HRE2^{3xHA}	[24]	*35S:HRE2*^{3xHA}
**Oligonucleotides**		
qPCR_UCP1_F: ATTCTGGAGCGCTGAATGCT	N/A	N/A
qPCR_UCP1_R: ATCCTGCTCCCAGTCCAGAT	N/A	N/A
qPCR_ADH1_F: CCCGGGGTTGTGGAAAAGTA	N/A	N/A
qPCR_ADH1_R: CCCATGGTGATGATGCAACG	N/A	N/A
qPCR_PGB1_F: AGGGAAAGTTACGGTGAGGG	N/A	N/A
qPCR_PGB1_R: TGCATACTTGGCCACCTCAA	N/A	N/A
qPCR_CML38_F: AAGCCCTTTCCCCTATTTCTCA	N/A	N/A
qPCR_CML38_R: CTCCGGCTGAATCTTCCCTC	N/A	N/A
qPCR_ACO1_F: ACCTCAGATGCAGATTGGGAAAGC	N/A	N/A
qPCR_ACO1_R: CCATCGTCTTGCTGAGTTCCTCTG	N/A	N/A
qPCR_PDC2_F: CCCCAAATCCGCAGTAGAGT	N/A	N/A
qPCR_PDC2_R: CCTCAAGGGGACACACATTT	N/A	N/A
qPCR_SRO5_F: CTTGGACCTCAAGTTCTTTTC	N/A	N/A
qPCR_SRO5_R: CGCAGCTTCCAGATTCAGAG	N/A	N/A

(*Continued on next page*)

Continued

REAGENT or RESOURCE	SOURCE	IDENTIFIER
qPCR_LBD41_F: TGAAGCGCAAGCTAACGCA	N/A	N/A
qPCR_LBD41_R: ATCCCAGGACGAAGGTGATTG	N/A	N/A
qPCR_PCO1_F: ATTGGGTGGTTGATGCTCCAATG	N/A	N/A
qPCR_PCO1_R: ATGCATGTTCCCGCCATCTTC	N/A	N/A
qPCR_PCO2_F: CTTCGAGCCGTTTTGGATGA	N/A	N/A
qPCR_PCO2_R: ACGTCACTAACGGAGATCGTCC	N/A	N/A
qPCR_DIC3_F: AATCTTCCCGTGAAACCTTAC	N/A	N/A
qPCR_DIC3_R: AAGGAAATGCTGCCGATGAG	N/A	N/A
qPCR_NDB4_F: GCCAGTGGCTTTGGTACTC	N/A	N/A
qPCR_NDB4_R: CTGCTAGAGTCACGGCCAA	N/A	N/A
Genotyping_UCP1_F: GACGAAGATGTGAAGTAGACC	N/A	N/A
Genotyping_UCP1_R: CAAAGAGAAGATACATGTTG	N/A	N/A
Genotyping_RAP2.2_F: ATGACAACATTGGGATGCAAC	N/A	N/A
Genotyping_RAP2.2_R: TTTCTTGGCATATGCTGAACC	N/A	N/A
Genotyping_RAP2.3_F: ATGTGTGGCGGTGCTATTATT	N/A	N/A
Genotyping_RAP2.3_R: TTACTCATACGACGCAATGAC	N/A	N/A
Genotyping_RAP2.12_F: CTCAGCTGTCTTGAACGTTCC	N/A	N/A
Genotyping_RAP2.12_R: TGGCTACTCCTGAATGCAAAC	N/A	N/A
Genotyping_HRE1_F: ACCGCGGGTTAAAATCTAGTG	N/A	N/A
Genotyping_HRE1_R: TTCAGCTGTGTTGAAAGTCCC	N/A	N/A
Genotyping_HRE2_F: TTGCAAAAGGTTATAGAGCACAC	N/A	N/A
Genotyping_HRE2_R: CGACGGTGTTTAGTGTGTTTG	N/A	N/A
Genotyping_PRT6_F: GGAGTTTTCTATGTCCAGTGAGAGTTT	N/A	N/A
Genotyping_PRT6_R: GTCTCCAATGACACGTTCACTTGTCT	N/A	N/A
Recombinant DNA		
PGWB14:UCP1	This study	35S:UCP1
Software and algorithms		
ImageJ	NIH – public domain	https://imagej.nih.gov/ij/download.html
Prism Graphpad v.8	Prism	Graphpad

RESOURCE AVAILABILITY

Lead contact

Further information and requests for resources and reagents should be directed to and will be fulfilled by the Lead Contact, Michael J. Holdsworth (michael.holdsworth@nottingham.ac.uk).

Materials availability

Materials generated in this study are available from the Lead Contact upon request. Distribution of transgenic lines is governed by the appropriate material transfer agreements (MTAs) and availability of seed material is dependent on provision of appropriate import permits acquired by the receiver.

Data and code availability

This study did not generate any unique datasets or code.

EXPERIMENTAL MODEL AND SUBJECT DETAILS

A. thaliana genetic materials including *ucp1*, *rap2.2 rap2.3*, *35S:MC-^{HA}GUS erfVII*, and *prt6-1* were described before,[27,28,30,36] except for UCP1ox lines (*35S:UCP1*) that were generated in this study. All mutants and transgenics are in the Col-0 accession (Wild Type, WT). For general growth plants were incubated in growth rooms under 120 μmol m^{-2} S^{-1} 12 h light at 21°C 12 h dark at 15°C in a mixture of Levington M3 compost (57%), Medium grade 2-5 mm vermiculite (29%) and Medium grade 2-5 mm perlite (14%). Growth conditions for specific experiments are given below in the Analysis of plant growth and response to stresses section.

METHOD DETAILS

Plant transformation

The *A. thaliana UCP1* coding sequence was amplified from Col-0 cDNA, recombined into pCR8GW (Invitrogen, Carlsbad, CA, USA), and then into pGWB14.[50] The construct was introduced into *Agrobacterium tumefaciens* (strain GV3101) that then used to transform *A. thaliana 35S:Met-Cys-^{HA}GUS*[21] using standard protocols.[51] Three transgenic events overexpressing UCP1 in the *35S:Met-Cys-^{HA}GUS* background, hereafter named as UCP1ox E#1, E#4 and E#6, were selected based on the *UCP1* expression level for further analysis. Oligonucleotide primers used for genotyping are shown in the key resources table.

Analysis of plant growth and response to stresses

Unless indicated, Petri plates containing half-strength Murashige and Skoog (MS) medium (pH 5.7) supplemented with 1% (w/v) agar were used in all assays.[52]

ABA

Seeds were sown on Petri plates supplemented with abscisic acid (1 μM ABA), and incubated in the dark for 96 h at 4°C. The plates were transferred to a growth chamber under a long-day photoperiod (16 h L, 8 h D).[52] Radicle emergence and greening of the cotyledons were scored.

Salt

Seeds were sown on Petri plates on half-strength MS medium pH 5.7 containing 1% (w/v) agar, stratified for 96 h at 4°C and grown in vertical position in constant light conditions for 3 days, when seedlings were transferred to plates containing half-strength MS supplemented with 150 mM NaCl for 7 days. Seedlings were then transferred back to fresh half-strength MS medium plates and after 5 days seedling survival was scored. For experiments performed at adult stage, plants were grown under neutral-day photoperiod (12 h L: 12 h D) and treatment started 3 weeks after germination of seeds sowed directly on soil.[21] Plants were watered with NaCl solution (150 mM) or water twice over 10 days in the case of plants used for cell damage assay. Irrigation with NaCl solution continued for 30 days in total before pictures were taken. Salt accumulation in the soil was avoided by allowing excess irrigation water to drain out of the pots.

Cold

Seeds were sown on half-strength MS medium, pH 5.7 containing 1% (w/v) agar, cold-treated for 96 h at 4°C, grown in vertical plates in constant light conditions for 12 days. The seedlings were transferred to growth-rooms in a long-day photoperiod (16 hL, 8 hD). Cold treatment was carried out using plants grown under long-day photoperiod for 21 days, after which they were transferred to a growth room with the same light intensity, but at 4°C. Plants were kept for 0, 12, 24, 48 and 96 h at 4°C, and the leaves were collected and frozen in liquid nitrogen.

Submergence

Seeds were sown on half-strength MS medium, pH 5.7 containing 1% (w/v) agar, stratified for 96 h at 4°C and grown in vertical plates at constant light conditions for 7 days. Approximately 50 seedlings were submerged in H_2O in an Eppendorf tube for 0, 10, 30, 60 and 120 min in the dark. Seedlings were removed from the tube and snap frozen in liquid nitrogen. The same number of seedlings were maintained in a humidity chamber for 0, 10, 30, 60 and 120 min in the dark as controls.

Hypoxia

Seeds were sown on quarter-strength MS medium, pH 5.7 containing 1% (w/v) agar, stratified for 96 h at 4°C and grown in vertical plates at short day conditions for 4 days. The opened plates were placed in a dark sealed chamber with constant flow of N_2 gas (4L/min) for 4 h, then closed and returned to the growth chamber. After 4 days the primary root tips were scored based on continued growth (survived).[23] Control untreated plates were kept in an opened dark chamber at normoxia for the same amount of time as treated plants.

Chemical treatment of leaf discs

Seeds were sown on half-strength MS medium, pH 5.7 containing 1% (w/v) agar, stratified for 96 h at 4°C and grown in vertical plates at neutral day conditions for 7 days. Then the seedlings were transferred to a soil and grown under neutral day conditions for an additional 3 weeks. Leaf discs were excised using a hole punch and incubated in quarter-strength MS medium, pH 5.7 separately supplemented or not with ADP (100 μM), GTP (100 μM), aspartate (5 mM), glutamate (5 mM), ethanol (0.1% v/v), HNE (in 0.1% ethanol v/v) (30 μM). Leaf discs were vacuum infiltrated for 5 min and incubated in the dark for 30 min. Then leaf discs were removed from the medium, washed three times in 100 mM sodium phosphate buffer (pH 7.0), the excess water was quickly removed in a paper towel and the discs frozen in liquid nitrogen.

SHAM and Antimycin A treatments

Transgenic *35S:MC-^{HA}GUS* or *35S:HRE2^{3xHA}* seedlings were grown in a Weiss Technik fitotron SGC 120 biological chamber under long day conditions (16 h light/ 8 h dark). For SHAM treatment, seedlings were grown in Levington F2 compost, vermiculite, and perlite mix (4:2:1 v/v) After 4 weeks, rosettes were randomized in trays and sprayed with solutions containing either 2% (v/v) ethanol, or 2% ethanol supplemented with 10 mM salicylhydroxamic acid (Sigma; S607). Leaves were then harvested and flash frozen in liquid nitrogen at time points indicated. For antimycin A treatment, seed were surface sterilized using 10% bleach and sown on square plates containing ½ MS medium pH 5.7 with 1% (w:v) agar. Ten d after germination, plates were then sprayed with solutions containing either 2% ethanol or 2% ethanol supplemented with 50 μM Antimycin A (Sigma; A8674). Whole seedlings were then harvested and flash frozen at the time points indicated.

Gene expression analyses

qRT-PCR

Total RNA was isolated from mature leaves (from 28 d-old plants grown in soil) or seedlings (age indicated for each experiment) using a RNAeasy Plant Mini Kit (QIAGEN) or a Nucleospin TriPrep (Macherey-Nagel) depending on the subsequent application. The RNA was used for first-strand cDNA synthesis using a Revertaid First Strand cDNA Synthesis kit (Fermentas, Waltham, MA, USA) according to the manufacturer's protocol. Real-time PCR was performed using the ABI PRISM 7500 system (Applied Biosystems, Foster City, CA, USA) with SYBR Green dye (Applied Biosystems). The reactions were performed at least in technical triplicate with three biological replicates and the results were expressed relative to the expression levels of a housekeeping gene in each sample using the $2^{-\Delta\Delta}C_T$ method. Oligonucleotide primers are shown in the key resources table.

Measurement of ion leakage

Cell damage was determined by measuring ion leakage as described.[21] Leaf disks of 0.6 cm^2 from 24 leaves were excised using a hole punch. Disks were rinsed briefly with water and floated on 5 mL of double distilled water for 6 h at room temperature. The conductivity of the water was measured using a conductivity meter (SevenGo, Mettler-Toledo, Columbus, OH, USA).

NO detection by fluorescence microscopy

Endogenous NO levels were measured as previously described[53] by immersing 9-day-old seedlings in 10 mM Tris-HCl (pH 7.4) containing 10 mM DAF-2 DA (4,5-diaminofluorescein diacetate, Sigma-Aldrich). Seedlings were shaken gently for 15 min in the dark, and subsequently washed for 20 min in 10 mM Tris-HCl (pH 7.4). The seedlings were visualized using a Leica DM5000B fluorescence microscope with excitation at 488 nm and emission 520 nm. NO intensity was determined by selecting equal areas of the same root zone and analyzing with Fiji software.[28] The tissue autofluorescence was subtracted from all of the measured samples.

Analysis of protein abundance by western blotting, and GUS enzyme activity

Protein extracts were prepared by grinding the tissue to a fine powder in liquid nitrogen and extracted using a buffer containing 50 mM Tris-HCl, pH 7.5, 150 mM NaCl, 5 mM $MgCl_2$, 1 mM EGTA, 10% (v/v) glycerol, 0.05% IGEPAL and 1x complete protease inhibitor cocktail (Roche, Basel, CH).[24] Total protein content in samples was quantified by Bradford protocol against a BSA standard curve. Total protein was separated by SDS–PAGE and transferred to nitrocellulose membrane (Immune-Blot PVDF, Biorad, Hercules, CA, USA) by electroblotting. Page Ruler Pre-Stained Protein Ladder (Invitrogen, Carlsbad, CA, USA) was loaded as a reference for protein size. Membranes were probed with anti-HA (Sigma-Aldrich, St. Louis, MO, USA), 1:2,500 (v/v) dilution; anti-actin (Agrisera, Vannas, SWE), 1:5,000; anti-UCP1 (Agrisera, Vannas, SWE), 1:2,000 and anti-ADH (Agrisera, Vannas, SWE), 1:2500 primary antibodies. The primary antibodies were detected using the following secondary antibodies: (goat) anti-rabbit IgG HRP conjugate (Invitrogen, Carlsbad, USA) at 1:10,000 dilution or (goat) anti-mouse IgG HRP conjugate (Invitrogen, Carlsbad, USA) at 1:10,000 dilution. Signal was detected using Pierce ECL Western Blotting Substrate (ThermoFisher, Waltham, MA, USA). GUS histochemical staining was carried out by incubating tissues in 100 mM phosphate buffer pH 7.0, containing potassium ferrycyanide (1 mM), potassium ferrocyanide (1 mM), Triton X-100 (0.1% v/v) and X-Gluc (1 mM). The samples were incubated at 37°C and photographed.

Western blot analysis of SHAM effects

Plant tissues were ground using a pestle and mortar in liquid N_2 and proteins were extracted using 150 mM Tris-HCl, pH 7.5, 150 mM NaCl, 5 mM EDTA, 2 mM EGTA, 5% (v/v) glycerol, 10 mM ditriothreitol (DTT), 0.5% Triton X-100 and protease inhibitor cocktail (Sigma-Aldrich; S8830) at a ratio of 1:3 w:v. Samples were run on 10% v:v poly-acrylamide gels and transferred to PVDF membranes overnight at 4°C. Membranes were blocked using 5% w:v milk (Marvel) in TBST, and probed with anti-β-glucuronidase (1:2000 dilution) (Sigma; G5420) and anti-Rabbit-HRP (1:2000 dilution) (Sigma; A0545). Signal was then detected using PierceTM ECL Western Blotting Substrate (Thermo Scientific; 32106)

QUANTIFICATION AND STATISTICAL ANALYSIS

All experiments were performed at least three times. Vertical lines represent standard deviation in all graphs. Statistical comparisons were conducted with RStudio v4.0.3 software. All collected data was evaluated for normality by the Shapiro-Wilk test and for homogeneity of variances by Levene's test. Data transformation was applied when necessary. For statistical comparisons, the one-or two-way Analysis of Variance (ANOVA) with Tukey's multiple comparisons test was used, employing the following factors when applied: genotype, treatment or tissue and the interaction. Significant differences (alpha < 0.05) are denoted with different letters. A t test was applied for Figure S2A because a large variance in gene expression was observed due to the discrepant profile of genotypes, hampering genotype differentiation by multiple comparison tests.

Current Biology, Volume *32*

Supplemental Information

Mitochondrial retrograde signaling through UCP1-mediated inhibition of the plant oxygen-sensing pathway

Pedro Barreto, Charlene Dambire, Gunjan Sharma, Jorge Vicente, Rory Osborne, Juliana Yassitepe, Daniel J. Gibbs, Ivan G. Maia, Michael J. Holdsworth, and Paulo Arruda

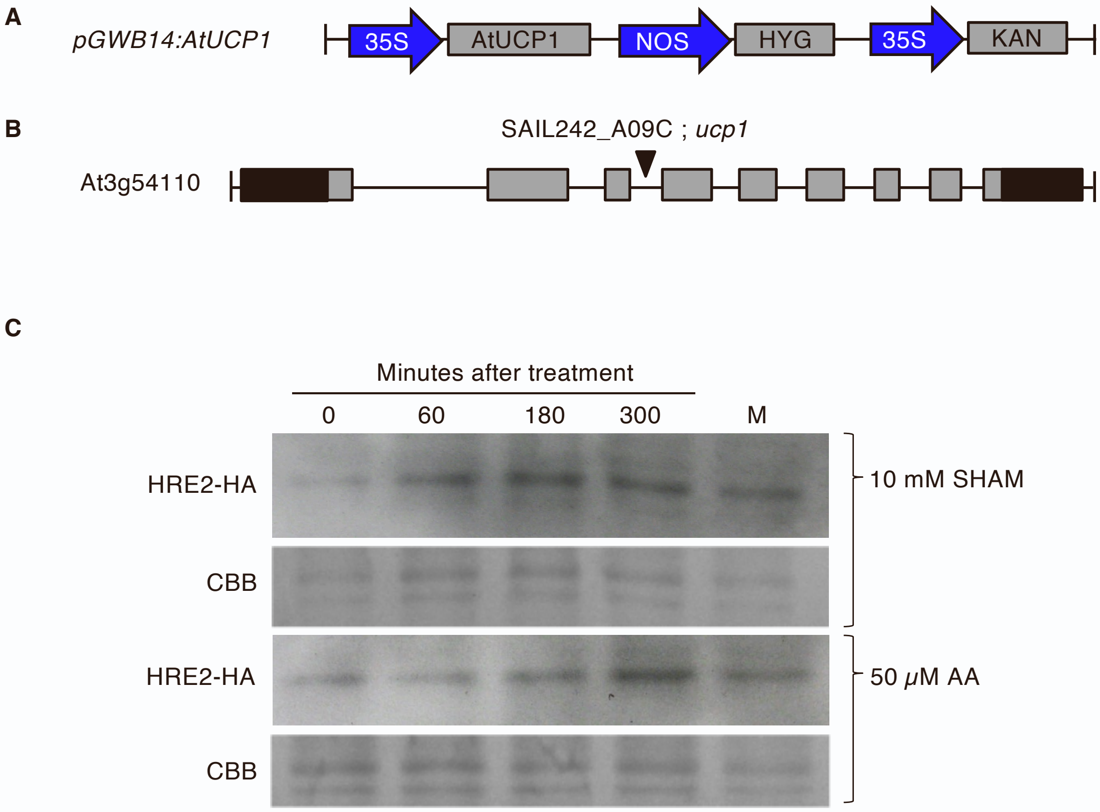

Figure S1. Diagrammatical representation of *35S:UCP1* construct transformed into *A. thaliana*, and *ucp1* mutant, and influence of mitochondrial inhibitors on stability of HRE2^{3xHA}. Related to Figure 1.
A. The *UCP1* coding sequence was recombined into the PGWB14 plasmid vector under the control of the 35S constitutive promoter and transformed into *35S:MC-HAGUS* line. **B.** A SAIL insertional knockdown for UCP1 (At3g54110) (*ucp1*). Grey boxes indicate coding regions, black untranslated regions of transcripts, blue arrows indicate promoters. **C.** Western blot analysis of HRE2^{3xHA} abundance following treatment with SHAM or Antimycin A (AA), M indicates mock samples sprayed with 2% ethanol only. CBB, Coomassie Brilliant Blue loading control.

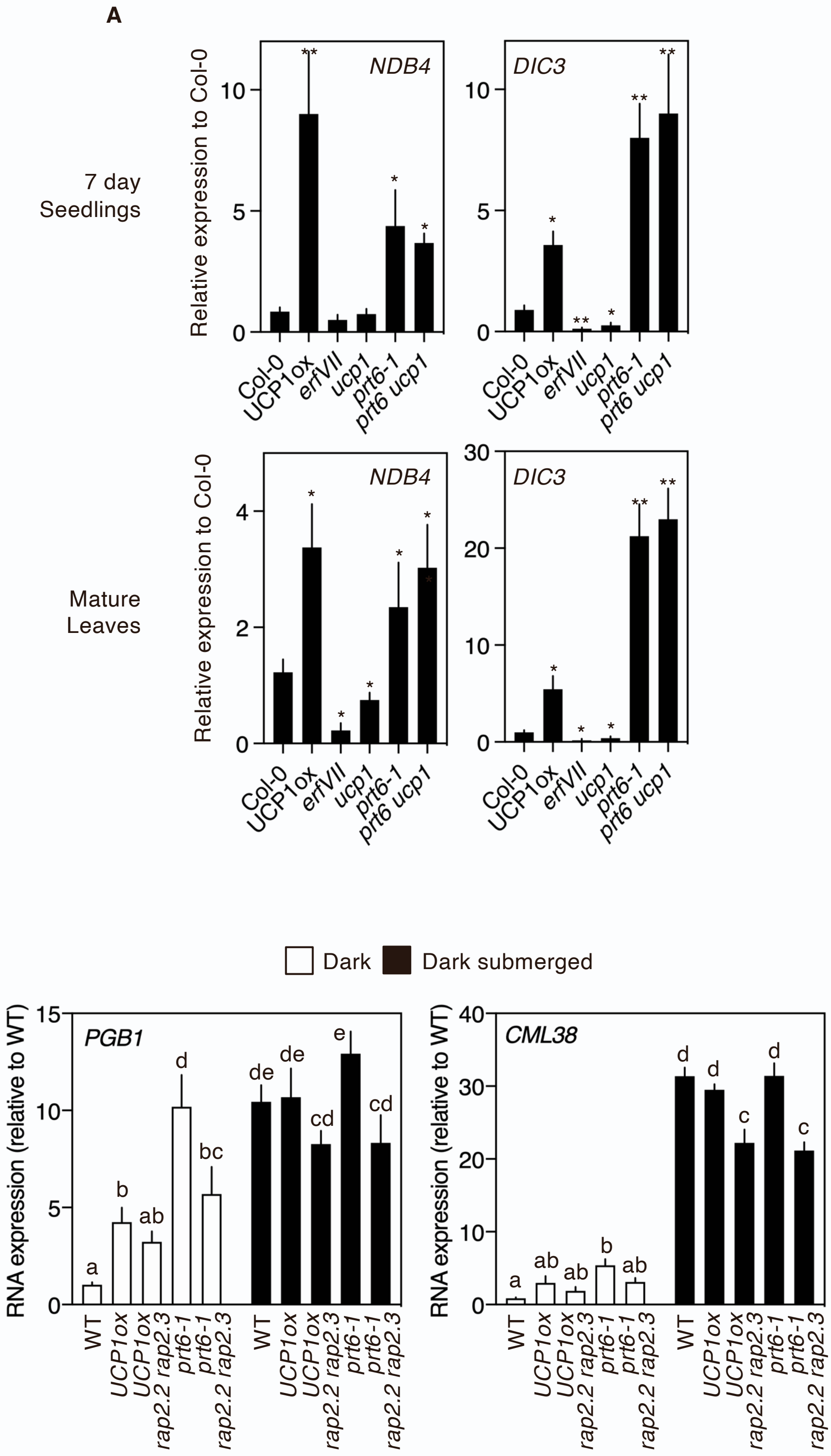

Figure S2. Expression of canonical genes of mitochondrial retrograde signalling and hypoxia response in seedlings/leaves and dark/dark+submergence. Related to Figure 2.

A. RNA expression for 7-day-old light grown seedlings for mitochondrial marker transcripts from nuclear genes *NDB4* and *DIC3*. T-tests were applied with comparison to Col-0 in each case because a large variance in gene expression was observed due to the discrepant profile of the genotypes analysed, hampering genotype differentiation by multiple comparison tests. * $p< 0.05$, ** $p< 0.01$.

B. RNA expression for 7-day-old seedlings grown in long days in control (1 h dark) or 1 h submerged+dark for WT, mutants and UCP1ox combinations.

Error bars indicate SD, letters one-way ANOVA, Tukey's test.

Figure S3, Related to Figure 3.

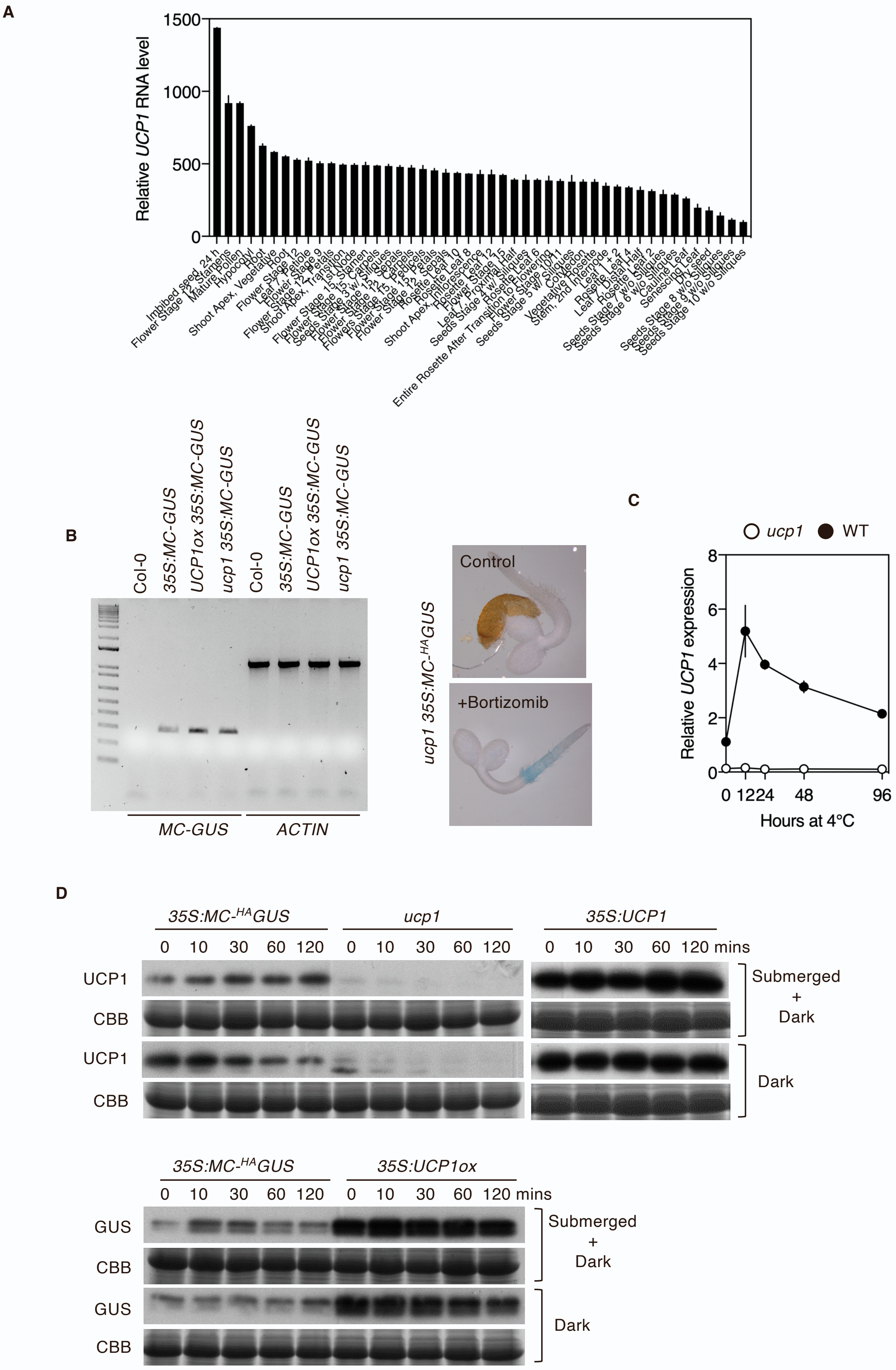

Figure S3. Gene expression and UCP1/^{HA}GUS stability. Related to Figure 3.

A. Relative levels of *UCP1* RNA expression during development in *A. thaliana* (data obtained from Bio Array Resource).

B. Semi quantitative rtPCR analysis of *MC-GUS* and *ACTIN* RNA expression in seedlings of mutant combinations and histochemical staining of GUS activity in *ucp1* seedlings in the absence (control) or presence of Bortezomib (20 mM treatment for 3h) .

C. Expression of *UCP1* RNA in WT (Col-0 *35S:MetCys-^{HA}GUS*) and *ucp1* following transfer to cold (4C).

D. UCP1 and ^{HA}GUS protein accumulation in *35S:Met-Cys-^{HA}GUS*, *UCP1ox* and *ucp1* subjected to 0, 10, 30, 60 and 120 min of submergence in water in the dark or only in the dark. CBB Coumassie Brilliant Blue.

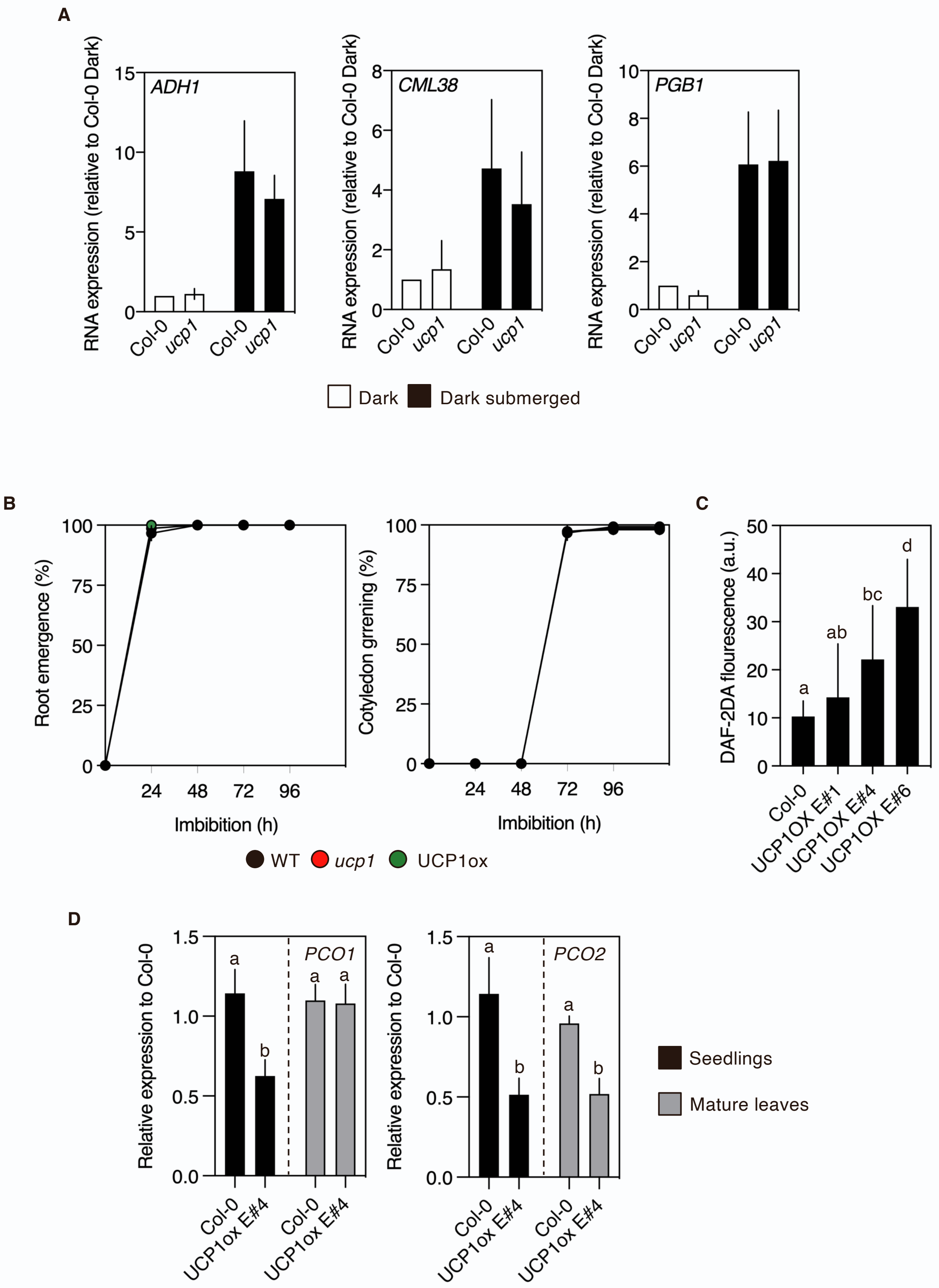

Figure S4. Gene expression, germination and NO accumulation. Related to Figure 4.

A. RNA expression for 7-day-old seedlings grown in continuous light in control (1 h dark) or 1 h submerged+dark for WT and *ucp1*.

B. Germination (root emergence) and establishment (cotyledon greening) of WT, *ucp1* and UCP1ox on 1/2MS media without ABA. Error bars indicate SD.

C. NO levels (measured as DAF-2DA fluorescence, arbitrary units) for WT and UCP1ox transgenic lines. Error bars indicate SD, letters one-way ANOVA, Tukey's test.

D. Expression of transcripts for *PCO1* and *PCO2* in seedlings or mature leaves of Col-0 and UCP1ox.
